# Increasing topical sunscreen use in German outdoor workers: results of a non-randomised controlled intervention study

**DOI:** 10.1186/s12889-026-29422-6

**Published:** 2026-09-23

**Authors:** Anja Dick, Michaela Ludewig, Christoph Skudlik, Ivone Jakasa, Sanja Kezic, Florentine de Boer, Henk van der Molen, Swen Malte John, Marc Rocholl

**Affiliations:** 1 Department of Dermatology, Environmental Medicine and Health Theory, Institute for Health Research and Education, Osnabrück University, Am Finkenhügel 7a, Osnabrück, Lower Saxony 49076 Germany; 2 Peter Greven Physioderm GmbH, Euskirchen, North Rhine Westphalia Germany; 3 Department of Health Sciences, University of Applied Sciences Bochum, Bochum, North Rhine Westphalia Germany; 4 Institute for Interdisciplinary Dermatological Prevention and Rehabilitation (iDerm) at Osnabrück University, Osnabrück, Germany; 5 Laboratory for Analytical Chemistry, Department of Chemistry and Biochemistry, Faculty of Food Technology and Biotechnology, University of Zagreb, Zagreb, Croatia; 6 Department Public and Occupational Health, Amsterdam UMC location University of Amsterdam, Amsterdam Public Health Research Institute, Amsterdam, The Netherlands; 7 Niedersächsisches Institut für Berufsdermatologie (NIB), Osnabrück University, Osnabrück, Lower Saxony Germany

**Keywords:** Sunscreen use, Occupational sun exposure, Outdoor workers, Skin cancer prevention, Workplace intervention, Health education

## Abstract

**Background:**

Occupational exposure to solar ultraviolet radiation (UVR) is a major environmental risk factor for skin cancer. Outdoor workers accumulate high lifetime UVR exposure and therefore face an increased risk of developing actinic keratoses and skin cancer. Despite established recommendations for sun protection, adherence among outdoor workers remains low. Improving access to sunscreen and providing targeted health education may help reduce barriers to sun-protective behaviour among outdoor workers. Therefore, this study evaluated the effectiveness of a workplace intervention designed to increase sunscreen use among outdoor workers.

**Methods:**

A non-randomised controlled intervention study was conducted among outdoor workers in Germany during one sun-exposure season (April–September 2025). Participants were assigned to either an intervention group or a control group. The intervention consisted of facilitated access to different sunscreen products combined with a brief structured health education on UVR risks and correct sunscreen use. Sunscreen use and sunburn occurrence were assessed using a questionnaire at baseline (T0), after three months (T1), and after six months (T2). The primary outcome was the proportion of workers reporting sunscreen use at least “sometimes”. Longitudinal changes were analysed using generalised linear mixed models.

**Results:**

A total of 234 outdoor workers were enrolled, with 117 participants in each group. After six months, 97 participants in the intervention group and 86 in the control group completed the study. At baseline, 54.7% of participants in both groups reported sunscreen use in general at least sometimes. At T2, this proportion increased to 83.5% in the intervention group compared with 52.3% in the control group. The number of reported sunburns decreased over time in both groups, with a stronger reduction observed in the intervention group; however, the group × time interaction did not reach statistical significance.

**Conclusions:**

A simple workplace intervention consisting of facilitated sunscreen provision and brief health education was associated with a substantial increase in sunscreen use over the six-month follow-up period. This low-threshold approach represents a feasible and scalable strategy for occupational skin cancer prevention.

**Trial registration:**

The study was approved by the Ethics Committee of Osnabrück University and registered in the German Clinical Trials Register (DRKS00035178; registration date: 10 October 2024).

**Graphical Abstract:**

**Figure:**
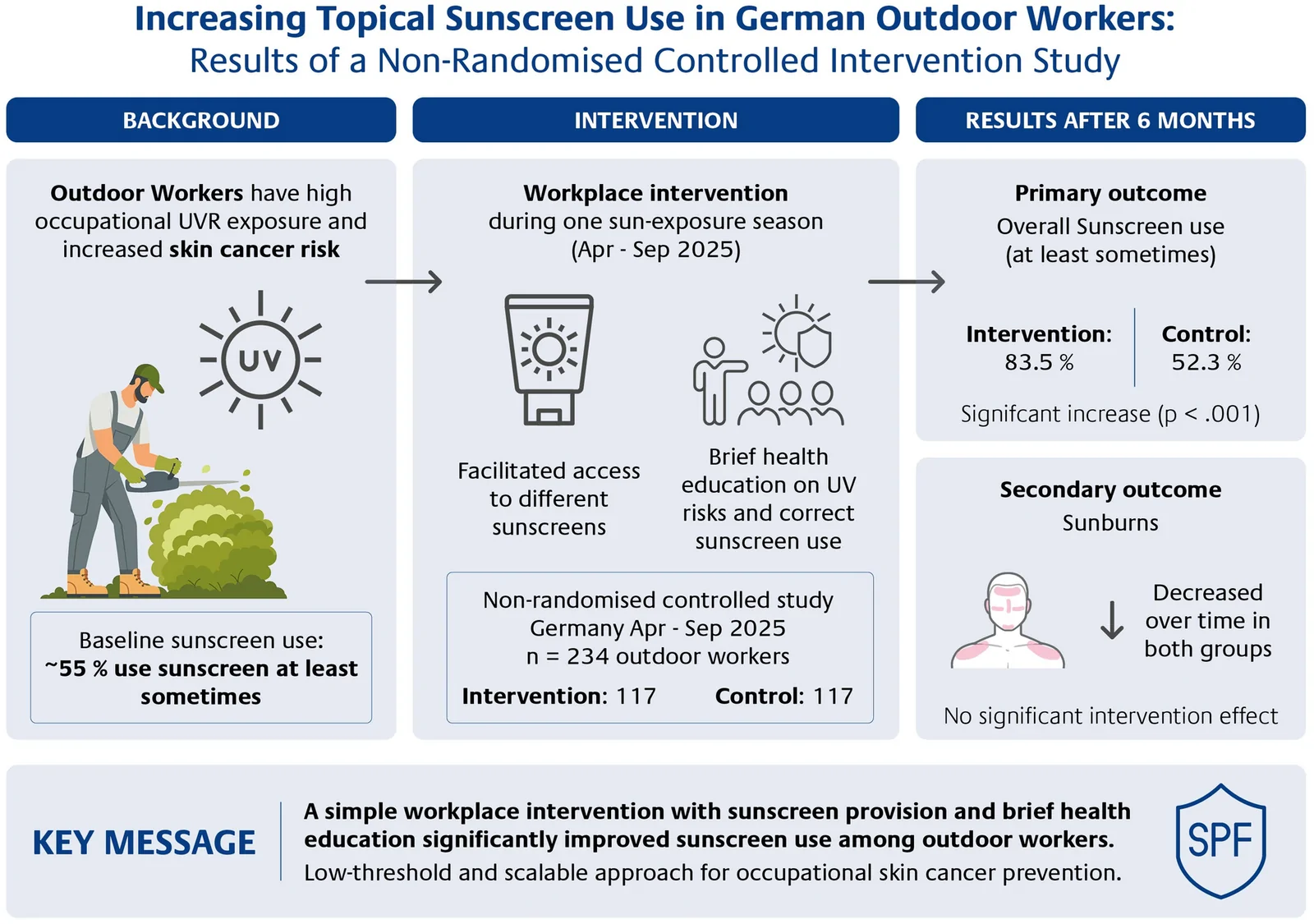

**Supplementary Information:**

The online version contains supplementary material available at https://doi.org/10.1186/s12889-026-29422-6.

## Background

Occupational exposure to solar ultraviolet radiation (UVR) is a major environmental risk factor for skin cancer and contributes substantially to the global burden of UVR-related skin diseases. Outdoor workers accumulate high lifetime UVR doses and are at least twice as likely to develop keratinocyte carcinoma (KC) – including actinic keratosis (AK), squamous cell carcinoma (SCC), and basal cell carcinoma (BCC) – compared to the general population [1–4]. Globally, an estimated 1.6 billion individuals, corresponding to 28.4% of the working-age population, were exposed to solar UVR at work in 2019, highlighting the widespread nature of this occupational hazard [2]. Occupational groups at particular risk include workers in construction, agriculture, forestry, and gardening [5]. In Germany, solar UVR-induced SCC and multiple AK are among the most frequently recognised occupational diseases [6]. Given their high incidence, chronic disease course, need for repeated dermatological treatment, and associated compensation payments, these conditions represent a considerable burden for the statutory accident insurance system, society, and the affected individuals. While evidence on the economic impact of occupational skin cancer remains limited, available data indicate that UVR-induced occupational skin cancer generates substantial direct and indirect costs, further underscoring its public health relevance [7].

Recommended sun protection strategies include the regular use of broad-spectrum sunscreen, protective clothing, and shade-seeking behaviour. However, adherence to these measures among outdoor workers remains consistently low [8, 9]. Observational studies suggest that sunscreen use in particular is insufficiently implemented during outdoor work, indicating a persistent gap between knowledge of sun protection and actual protective behaviour [10]. As sunscreen represents one of the most feasible protective measures in many occupational contexts, increasing its regular use may substantially contribute to reduce occupational UVR exposure. From a public health perspective, improving sunscreen use is a scalable and low-threshold component of comprehensive sun-protection strategies with potential relevance for population-level UVR prevention.

Interventions aimed at improving sun-protective behaviour have been developed to address this gap. Multicomponent approaches are considered particularly promising, as they combine educational components with structural or environmental support to facilitate behaviour change [11–13]. In occupational settings, this may include targeted health education together with the direct provision of sunscreen to reduce practical barriers to use. Despite growing interest in workplace sun-safety interventions, evidence remains limited regarding interventions that isolate sunscreen use as their primary target. Previous studies have typically evaluated multicomponent programmes that combine educational, organisational, and environmental measures, including workplace policies, shade provision, protective clothing, equipment, skin examinations, and role modelling [11–15]. Consequently, it remains unclear to what extent a pragmatic intervention centred on sunscreen provision and application alone can change behaviour across diverse occupational settings, highlighting a gap in current evidence.

Existing observational studies illustrate the magnitude of the behavioural gap. For example, a Canadian cohort study reported that only around 25% of outdoor workers regularly used sunscreen, while a Dutch study observed a baseline usage rate of 38% [10, 16]. These findings indicate that the majority of outdoor workers do not consistently apply sunscreen and highlight the need for effective workplace-based interventions to improve sun-protective behaviour.

While comprehensive sun-safety policies and organisational measures (e.g., shade provision, adaptive scheduling) remain the gold standard for occupational UVR prevention, there is a need for evidence on low-threshold, individual-level measures that can be readily implemented in diverse workplace settings. The present study therefore focuses on sunscreen provision and application as a complementary personal protective component, without intending to substitute broader occupational sun-safety strategies. Accordingly, the present study aims to evaluate the effectiveness of a workplace intervention designed to increase sunscreen use among outdoor workers. The intervention combined facilitated access to different sunscreen products with a brief structured health education. The primary outcome was the frequency of overall sunscreen use during a six-month follow-up period. By generating evidence from real-world occupational settings and focusing on a specific, readily implementable component of sun protection, this study aimed to address the limited evidence on sunscreen-focused workplace interventions in diverse European outdoor occupations and to clarify the potential contribution of facilitated sunscreen access and education within, rather than as a substitute for, comprehensive occupational sun-safety strategies.

## Methods

The study was conducted in accordance with the principles of the Declaration of Helsinki and was approved by the Ethics Committee of Osnabrück University, Germany (reference number: Ethik-37/2024). Written informed consent was obtained from all participants prior to study participation. The study design and methodology were described in detail in a previously published study protocol [17]. A brief summary is provided below. This report follows the Transparent Reporting of Evaluations with Non-randomised Designs (TREND) statement [18].

### Study design and setting

We conducted a non-randomised controlled intervention study among German outdoor workers. The observation period spanned one sun-exposure season (April - September 2025). The effectiveness of the intervention was assessed by self-reported sunscreen use by a structured questionnaire. Data collection occurred at three time-points: baseline (T0), after 3 months (T1) and after 6 months (T2) during on-site visits by the researchers.

### Participants and recruitment

Companies operating in outdoor-intensive sectors (e.g., gardening, landscaping, construction) were invited to participate. A total of 58 companies were enrolled, contributing 234 participants. Due to organisational constraints and the need to avoid contamination between workers within the same company, random allocation was not feasible. Companies were therefore assigned pragmatically to intervention or control conditions. Participants were required to be at least 18 years old and experienced regular, intensive exposure to solar UVR – one hour or more per day, as defined in the occupational health rule No. 13.3 from the Federal Institute of Occupational Safety and Health (AMR 13.3) [19]. Individuals with known allergies to any study product ingredients were excluded.

### Intervention

Each participant of the intervention group received an initial sunscreen kit containing four tubes of SPF 50 + sun cream, two bottles of SPF 50 sun spray, and one SPF 30 lip care sun stick. Providing different formulations (cream, spray and lip protection) was intended to increase acceptability and accommodate individual preferences. Details regarding the sunscreen products are described in the study protocol [17]. To maintain adequate sunscreen supply, a second identical kit was provided after three months. While these provisions were expected to be sufficient for the study duration, participants could request additional products as needed. Participants also received comprehensive health education, to promote consistent use of the study products in both occupational and private settings. The intervention included information on the risks associated with occupational UVR exposure and the importance of sunscreen use. Key educational components were the fundamentals of UVR, its adverse effects, UVR protection, as well as detailed instructions on the correct application of the provided sunscreen products. Participants were instructed to apply sunscreen before starting outdoor work, reapply it every two hours, use a sufficient amount of product, cover all uncovered skin areas, and follow product-specific dosing recommendations, including the two-finger rule for the sunscreen cream and 10 pump sprays per body zone for the sunscreen spray.

The health education was delivered in group or individual sessions, depending on the number of participants at each company, and lasted approximately 5–10 min. Additionally, all information was also provided in writing through the distribution of flyers, which are provided as Supplementary Material S1. To ensure comprehension among all participants, the educational materials were prepared using clear and accessible language. The control group received no intervention and continued usual practice. The use of alternative sunscreen or sun protection products was permitted in both groups.

### Questionnaire

The frequency of sunscreen use was asked in a questionnaire adapted according to Symanzik et al. [20], also including items on work status, UVR-related job characteristics, and the number of sunburns. The questionnaire was administered in German only, and German language proficiency was an eligibility criterion for study participation. Therefore, no language-related barriers were encountered during data collection. The questionnaire is provided as Supplementary Material S2. The scale regarding the questions about the frequency of use was adapted according to questionnaires of Peters et al. [10] and Keurentjes et al. [16] and employed a 5-point Likert scale (1 = never, 2 = rarely, 3 = sometimes, 4 = often, 5 = always), assessed separately for overall use, occupational use, and private use. Sunburn occurrences were assessed separately from sunscreen-use frequency. At T0, participants retrospectively reported the number of sunburns they had experienced during the preceding April – September season (2024). Sunburns were not classified according to whether they occurred in occupational or private settings. For the 2025 season, sunburn data were collected at T2, representing the April – September 2025 season.

### Power calculation

The primary outcome of the study was the proportion of outdoor workers who generally apply topical sunscreen with a frequency of “sometimes”, “often” or “always” at the end of the observation period (on a scale of never, rarely, sometimes, often, always). A Canadian study reported, that without intervention, 25% of outdoor workers used sunscreen regularly [10]. A Dutch study using a similar frequency score reported, without intervention, an application rate of 38% [16]. For this study, it was assumed that the proportion of outdoor workers in the control group who apply sun protection products at least “sometimes” is 30%. It was expected that in the intervention group, this proportion can be increased to 50%. With a sample size of 93 outdoor workers per group, a two-sample chi-square test with a 0.05 two-sided significance level would then have 80% power to detect this difference between the application frequencies (SAS V.9.4, PROC POWER). It was expected that not all outdoor workers will complete the study as planned. Considering a 20% rate of premature drop-outs, 117 outdoor workers were recruited per group. In total, 234 outdoor workers were included in this study.

### Statistical analysis

Descriptive statistics (means, standard deviations, and proportions) were calculated to summarise participant characteristics and outcome variables. Baseline differences between groups were assessed using chi-square tests for categorical variables and Mann-Whitney U tests for ordinal variables. Longitudinal changes in sunscreen use were analysed using generalised linear mixed models (GLMM), including group, time, their interaction, and sex as fixed effects, with a random intercept for participants. For these analyses, sunscreen use variables were dichotomised prior to modelling. Dichotomisation was performed a priori to align with the predefined primary endpoint and sample size calculation. Because sunburn counts showed substantial overdispersion, a GLMM with a negative binomial distribution and log link function was used. The model included group, time, their interaction, and sex as fixed effects, with a random intercept for participants. Statistical significance was set at *p* < .05. All analyses were conducted using IBM SPSS Statistics version 30.0.

## Results

### Study population

**Fig. 1:**
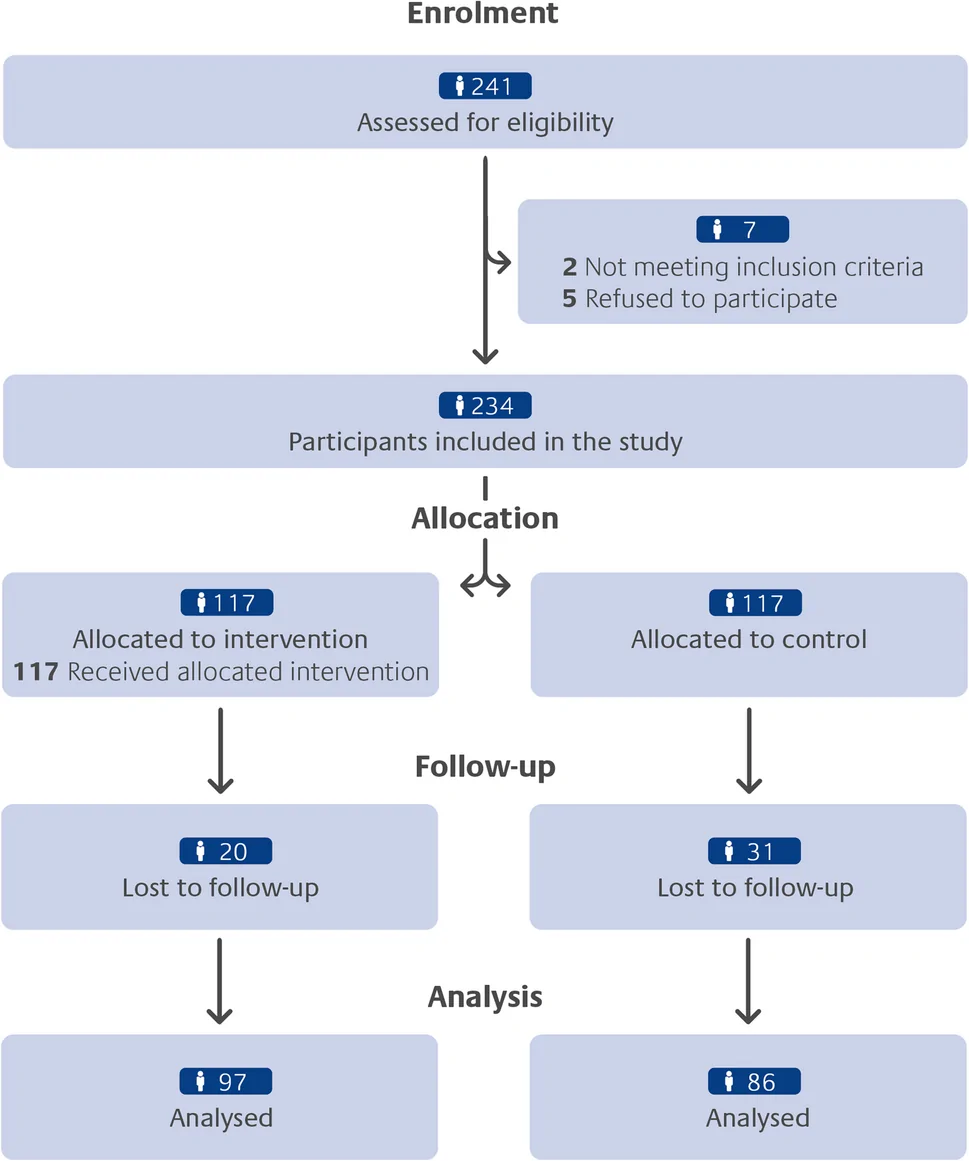
Flow diagram of participant recruitment, allocation, follow-up and analysis

The participant flow throughout the study is presented in Fig. 1. Baseline characteristics of the study population are summarized in Table 1. Baseline descriptive statistics refer to the full study population (*n* = 234). At study entry (T0), a total of 234 participants were enrolled and equally allocated to the intervention group (*n* = 117) and the control group (*n* = 117). At the first follow-up (T1), 101 participants in the intervention group and 89 participants in the control group remained in the study, corresponding to attrition rates of 13.7% and 23.9%, respectively. At the second follow-up (T2), 97 participants in the intervention group (cumulative attrition rate of 17.1% from baseline) and 86 participants in the control group (cumulative attrition rate of 26.5% from baseline) completed the questionnaires. Reasons for dropout were not systematically assessed. Potential explanations include absence during follow-up visits, changes in employment status, or loss of interest in participating. GLMM were fitted using all available observations across measurement occasions, thereby retaining participants with incomplete follow-up data in the longitudinal analyses. Most baseline characteristics did not differ significantly between groups. However, significant group differences were observed for sex distribution (*p* = .046), with the intervention group including a lower proportion of male participants (64.1% vs. 76.1%) and a higher proportion of female participants (35.9% vs. 23.9%) than the control group. Differences in occupational sector should be interpreted with caution due to small cell counts. Sex was included as a covariate in all primary models because baseline sex distribution differed significantly between groups.

The mean age of the sample at baseline was 40.5 years (SD = 14.0). The majority of participants were male (70.1%, *n* = 164) and female participants comprised 29.9% (*n* = 70). Regarding occupational sectors, participants were employed in construction (28.2%, *n* = 66), gardening and landscaping (18.8%, *n* = 44), education and social services (14.1%, *n* = 33), agriculture (12.0%, *n* = 28), public safety (6.8%, *n* = 16), industrial engineering (5.6%, *n* = 13), energy and environment (5.1%, *n* = 12), real estate (3.4%, *n* = 8), and other sectors (6.0%, *n* = 14). The “other” category comprised facility management, event management, field services, and logistics. Most participants reported working between 2 and 4 h (35.9%, *n* = 84) or more than 6 h (35.5%, *n* = 83) outdoors per day. Smaller proportions reported working 5–6 h (17.5%, *n* = 41) or less than 2 h (11.1%, *n* = 26) outdoors daily. Baseline characteristics of participants retained until T2 and those lost to follow-up were compared to assess potential attrition bias. No statistically significant differences were observed for age (40.9 ± 13.7 vs. 38.4 ± 15.0 years; *p* = .277), sex distribution (χ2 (1) = 1.76, *p* = .185), or daily outdoor exposure (χ2 (3) = 2.67, *p* = .446). However, occupational sector differed significantly between participants retained until T2 and those lost to follow-up (χ2 (9) = 34.41, *p* < .001), indicating that attrition varied across occupational sectors, although this comparison should be interpreted cautiously because several occupational categories contained small numbers.

**Table 1:** Baseline characteristics of participants

Characteristic	Intervention group (*n* = 117)	Control group (*n* = 117)	*p* value
Age			0.121
Mean (SD)	42.1 (15.0)	38.9 (12.8)	
Sex			0.046
Male *n* (%)	75 (64.1%)	89 (76.1%)	
Female *n* (%)	42 (35.9%)	28 (23.9%)	
Daily outdoor exposure			0.951
< 2 h *n* (%)	13 (11.1%)	13 (11.1%)	
2–4 h *n* (%)	44 (37.6%)	40 (34.2%)	
5–6 h *n* (%)	16 (13.7%)	25 (21,4%)	
> 6 h *n* (%)	44 (37.6%)	39 (33.3%)	
Occupational sector			< 0.001
Construction *n* (%)	40 (34.2%)	26 (22.2%)	
Gardening and landscaping *n* (%)	27 (23.1%)	17 (14.5%)	
Education and social services *n* (%)	24 (20.5%)	9 (7.7%)	
Agriculture *n* (%)	7 (6.0%)	21 (17.9%)	
Public Safety *n* (%)	1 (0.9%)	15 (12.8%)	
Industrial engineering *n* (%)	7 (6.0%)	6 (5.1)	
Energy and environment *n* (%)	0 (0.0%)	12 (10.3%)	
Real estate *n* (%)	6 (5.1%)	2 (1.7%)	
Others *n* (%)	5 (4.3%)	9 (7.7%)	

### Frequency of overall sunscreen use

**Fig. 2:**
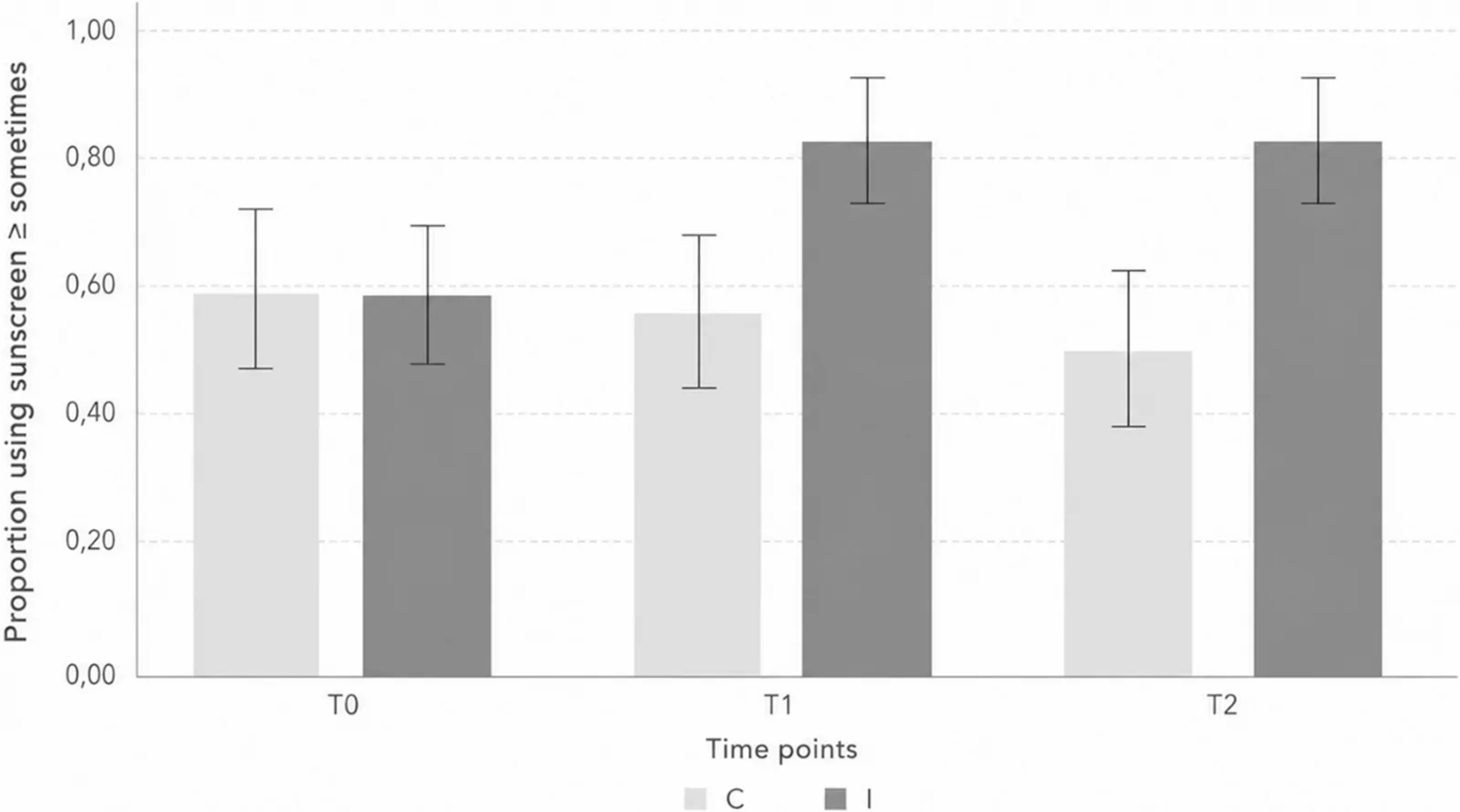
Estimated proportion of participants using sunscreen (≥ sometimes) over time (T0–T2) in the intervention (I) and control (C) groups. Error bars indicate 95% confidence intervals

At baseline (T0), 54.7% (128/234) of participants reported overall sunscreen use at least “sometimes” with no differences between the intervention and control groups (χ² (1) = 0.00, *p* = 1.00). Mean overall sunscreen use was also comparable between groups (intervention: M = 2.6, SD = 1.1; control: M = 2.7, SD = 1.0), corresponding approximately to use between “rarely” and “sometimes”. As illustrated in Fig. 2, overall sunscreen use increased markedly over time in the intervention group, whereas use in the control group was comparatively stable, showing a modest decrease from 55% at T0 to 52% at T2. This decline in the control group further contributed to the observed between-group difference in sunscreen use between the two groups at T2. This pattern is further reflected in the distribution of response categories (Table 2), showing a clear shift from lower to higher frequency categories in the intervention group over time. By T2, 83.5% (81/97) of participants in the intervention group reported sunscreen use of at least “sometimes”, compared with 52.3% (45/86) in the control group. A GLMM revealed a significant interaction between time and group (*p* < .001), indicating that the increase in sunscreen use over time was significantly greater in the intervention group than in the control group.

### Frequency of occupational sunscreen use

For occupational sunscreen use, 42.3% (99/234) of participants reported using sunscreen at work at least “sometimes” at T0 with no difference between the intervention and control groups (χ² (1) = 0.16, *p* = .691). Mean occupational sunscreen use was comparable between groups (intervention: M = 2.4, SD = 1.2; control: M = 2.3, SD = 1.2), corresponding approximately to “rarely”. By T2, 72.2% (70/97) of participants in the intervention group reported sunscreen use of at least “sometimes”, compared with 40.7% (35/86) in the control group. This difference was supported by a significant group × time interaction in the GLMM (*p* < .019), indicating a greater increase over time in the intervention group.

### Frequency of private sunscreen use

52.1% (122/234) of participants reported using sunscreen in private settings at least “sometimes” at T0 with no difference between the intervention and control groups (χ² (1) = 0.07, *p* = .794). Mean private sunscreen use was also comparable between groups (intervention: M = 2.7, SD = 1.1; control: M = 2.6, SD = 1.0), corresponding approximately to use between “rarely” and “sometimes”. By T2, 72.2% (70/97) of participants in the intervention group reported sunscreen use of at least “sometimes”, compared with 54.7% (47/86) in the control group. No significant group × time interaction was observed for private sunscreen use (*p* = .248). However, a significant main effect of time was found (*p* = .002), indicating changes in private sunscreen use across measurement occasions. In addition, a significant main effect of group was observed (*p* = .010), suggesting overall differences between intervention and control participants. Female participants reported higher private sunscreen use than male participants (sex effect: *p* < .001).

**Table 2:** Sunscreen frequency distribution for overall (general) sunscreen use in intervention group (I) and control group (C)

Sunscreen frequency	T0		T1		T2	
	I *n* (%)	C *n* (%)	I *n* (%)	C *n* (%)	I *n* (%)	C *n* (%)
Never to rarely (= 1–2)	53 (45)	53 (45)	18 (18)	28 (35)	16 (17)	41 (48)
Sometimes to always (= 3–5)	64 (55)	64 (55)	83 (82)	51 (65)	81 (83)	45 (52)

### Number of sunburns

**Fig. 3:**
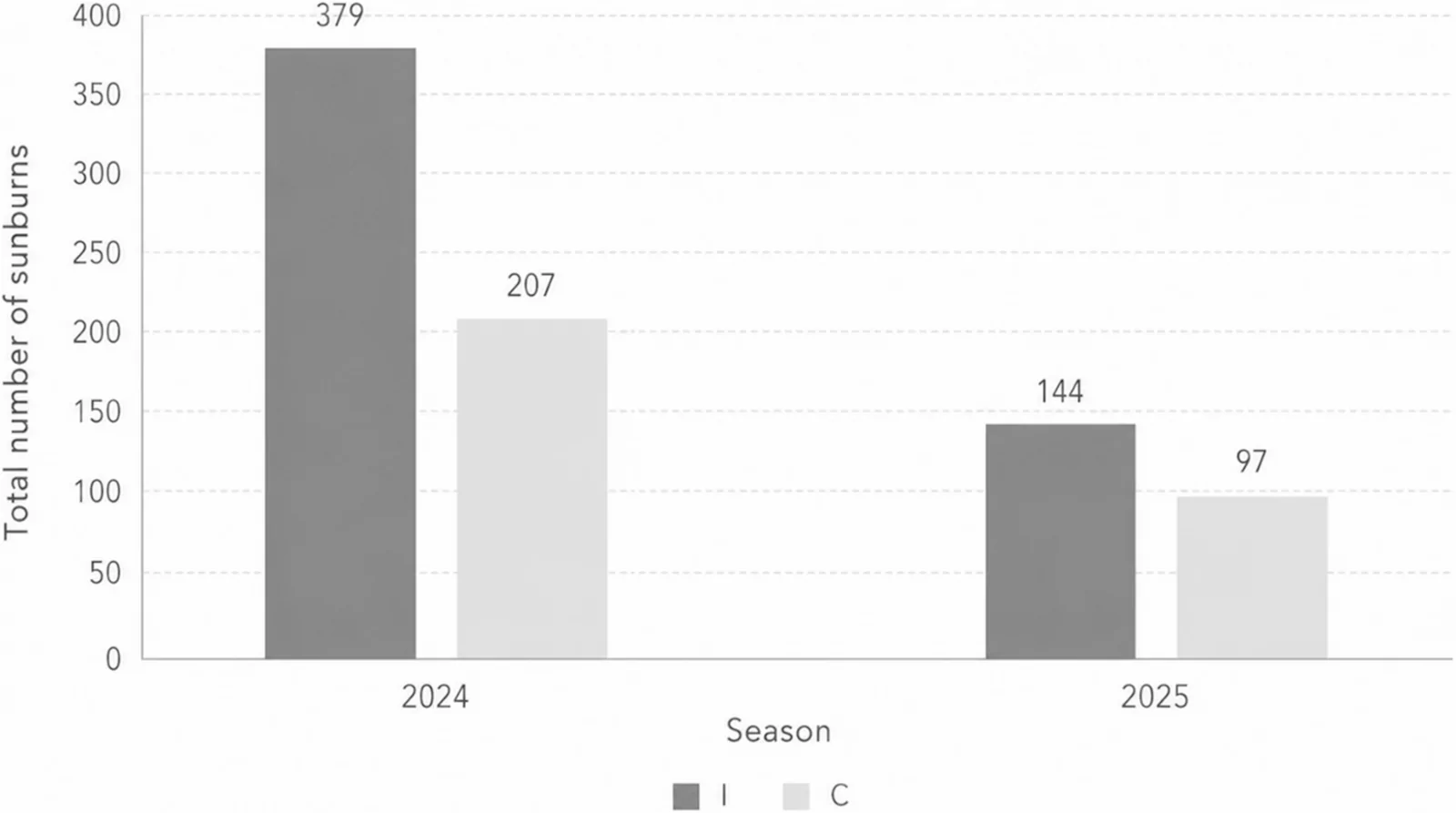
Total number of reported sunburns for the 2024 and 2025 seasons in the intervention (I) and control (C) groups. The 2024 values were collected retrospectively at T0 and should therefore be interpreted with caution

The total number of reported sunburns for the 2024 and 2025 seasons is presented in Fig. 3. The 2024 data were collected retrospectively at T0, before delivery of the intervention, and therefore represent a pre-intervention baseline measure that is particularly susceptible to recall bias. The intervention group reported more sunburns in the 2024 season than the control group (379 vs. 207). This imbalance cannot be attributed to the intervention. Baseline daily outdoor exposure did not differ between groups (*p* = .951), but the duration and intensity of UVR exposure during the 2024 season were not measured in sufficient detail to exclude chance differences in exposure. Sunburns were not distinguished according to occupational versus private setting. Because of the retrospective ascertainment of the 2024 season, the sunburn results are interpreted as exploratory and are not used as evidence of an intervention-specific clinical effect. For the 2025 season, sunburn data were collected at T2. To account for the count nature and overdispersion of the sunburn data, a GLMM with negative binomial distribution and log link was applied. A significant main effect of time was observed (*p* = .012), indicating an overall reduction in reported sunburns from 2024 to 2025. A significant main effect of group was also found (*p* = .007), with higher sunburn counts in the intervention group across measurement occasions. However, the group × time interaction was not statistically significant (*p* = .655), indicating that the reduction over time did not differ between intervention and control groups. Sex was not significantly associated with sunburn counts (*p* = .187).

## Discussion

This study evaluated the effectiveness of a pragmatic workplace intervention combining the provision of multiple sunscreen products with a brief health education among outdoor workers. The findings demonstrate that the intervention was associated with a statistically significant increase in sunscreen use throughout the six-month follow-up period. Participants in the intervention group showed a substantially larger increase in sunscreen use over time compared with the control group, as reflected by significant group × time interactions in the mixed-effects models.

These findings are consistent with previous research indicating that multicomponent interventions addressing both knowledge and structural barriers can effectively improve sun-protective behaviour in occupational settings [10–12]. However, the baseline situation in our study differed from assumptions underlying previous studies, as approximately 55% of participants already reported using sunscreen at least “sometimes” at baseline. Despite this relatively favourable starting point, the intervention was still associated with a marked increase in sunscreen use. The present study therefore extends existing evidence by demonstrating that a simple and easily implementable intervention can promote sun-protective behaviour under real-world occupational conditions.

The intervention did not result in a statistically significant intervention-specific reduction in the number of reported sunburns Although the number of reported sunburns decreased over time in both groups, the group × time interaction was not significant. These findings should be interpreted with caution because baseline conditions were not fully comparable between groups. Since significant baseline differences in sex distribution were observed, sex was included as a covariate in the primary analyses. The main findings remained unchanged, suggesting that the observed intervention effects were not solely explained by differences in sex distribution. In addition, the intervention group reported substantially more sunburns during the retrospectively assessed 2024 season than the control group, representing a baseline imbalance rather than an intervention effect. Several factors may explain the absence of a statistically significant intervention effect on sunburn occurrence. First, as previously noted, the 2024 data were collected retrospectively and are therefore subject to recall bias, which may have influenced the baseline comparison. Consequently, these findings are interpreted as exploratory and are not used as evidence of an intervention-specific clinical effect. Participants may have over- or underestimated the number of previous sunburns, potentially affecting comparisons between seasons. Moreover, the study did not distinguish between occupational and private sunburns, and detailed information on UVR exposure intensity during the 2024 season was not available. Second, repeated assessments may have increased awareness of sun protection among participants in both groups, thereby contributing to behavioural changes independent of the intervention itself. Similar measurement effects have been described previously and may partly explain improvements observed in control groups [21]. Third, participation in the study may have increased awareness of sun protection generally (Hawthorne effect), thereby influencing behaviour regardless of intervention allocation [22]. Moreover, sunscreen represents only one component of occupational UVR protection. According to the hierarchy of control measures, personal protective measures such as sunscreen are considered the final level of protection and should ideally complement technical and organisational measures, including the provision of shade, scheduling work outside peak UVR periods, and the use of protective clothing [23]. Because the present study focused specifically on sunscreen use, no conclusions can be drawn regarding changes in overall occupational sun-protective behaviour. This may also partly explain why increased sunscreen use did not translate into a measurable intervention-specific reduction in sunburn incidence.

Furthermore, sunburn occurrence is influenced by multiple factors beyond sunscreen use, including duration and intensity of UVR exposure, individual susceptibility, shade-seeking behaviour, and the use of other protective measures such as protective clothing, hats, and sunglasses. The present study focused primarily on sunscreen use and did not systematically assess changes in these additional sun-protective behaviours. Such behavioural changes may have contributed to the reduction in sunburns observed in both groups and may partly explain why increased sunscreen use did not translate into a statistically significant intervention-specific reduction in sunburn incidence. Similar patterns have been reported previously. For example, de Boer et al. observed a reduction in sunburn incidence in both intervention and control groups when evaluating a workplace-based intervention, which was partly attributed to environmental and contextual factors beyond the intervention itself [24].

A major strength of this study is the simplicity and feasibility of the intervention, which enhances its external validity and potential for transferability to other occupational contexts. The combination of direct sunscreen provision and brief, targeted health education addresses important barriers to sunscreen use, including product availability and limited awareness of occupational UVR risks. The intervention required relatively few resources and could be readily integrated into existing occupational health and safety structures, making it particularly relevant for small and medium-sized companies. Furthermore, the pragmatic implementation under real-world conditions enhances its external validity, practical applicability of the findings and potential for transferability to other occupational contexts. An additional strength of this study is the inclusion of participants from diverse occupational sectors. Unlike many previous studies that focused on specific industries, this heterogeneous sample enhances the external validity and transferability of the findings across different outdoor work settings. The longitudinal design and use of mixed-effects models additionally allowed appropriate analysis of repeated measurements while incorporating all available observations across time points.

Several limitations should be considered. First, the non-randomised design may have introduced selection bias and residual confounding. Although baseline differences in sex distribution were addressed statistically, unmeasured differences between groups may still have influenced the results. Second, allocation occurred at company level, raising the possibility of clustering effects resulting from shared working conditions, organisational safety culture, or similar occupational UVR exposure patterns. Although our mixed-effects models included a random intercept for participants to account for repeated measurements over time, we did not incorporate an additional company-level random intercept. Consequently, residual correlation between participants within the same company – due to shared safety cultures or exposure patterns – may not have been fully accounted for. This could potentially lead to an underestimation of standard errors and an overstatement of statistical significance. Future studies should consider multilevel models incorporating company-level random effects to account for this hierarchical data structure. Third, all outcomes were self-reported and therefore subject to recall bias and social desirability bias. Fourth, although mixed-effects models allow the inclusion of incomplete repeated-measures data under a missing-at-random assumption, differential attrition between groups may still have influenced the findings. The control group experienced a higher loss to follow-up than the intervention group, and attrition bias cannot therefore be completely excluded.

Overall, these findings suggest that improving access to different sunscreen products combined with brief health education may represent an effective and scalable strategy to promote sun-protective behaviour among outdoor workers. However, translating behavioural improvements into measurable clinical outcomes such as reduced sunburn incidence may require longer follow-up periods and more comprehensive assessment of sun-protective practices. Future studies should evaluate whether sustained improvements in sunscreen use and other protective behaviours ultimately translate into reductions in UVR-related skin disease outcomes.

## Conclusion

This non-randomised controlled intervention study demonstrates that a simple, low-threshold workplace intervention consisting of the provision of different sunscreens combined with brief health education may substantially improve sun-protective behaviour among outdoor workers. The intervention resulted in a significant increase in overall sunscreen use. Although sunburn incidence decreased over time in both groups, no statistically significant difference between the intervention and control groups was observed. This suggests that the behavioural improvements did not result in a clearly demonstrable intervention-specific reduction in sunburns within the study period.

Given its practicality and low implementation burden, this approach represents a feasible and scalable strategy for occupational skin cancer prevention. Integrating structured sunscreen provision into routine workplace health and safety measures may contribute to improved sun protection in outdoor occupations. Future studies with longer follow-up periods and more precise exposure assessment are warranted to evaluate the long-term clinical impact of such interventions.

## Supplementary Information

Supplementary Material 1.

Supplementary Material 2.

Supplementary Material 3.

## Data Availability

The data that support the findings of this study are available from the corresponding author upon reasonable request.

## Abbreviations

AK: Actinic keratosis
AMR: Occupational Health Rule (“Arbeitsmedizinische Regel”)
BCC: Basal cell carcinoma
DRKS: German Clinical Trials Register (“Deutsches Register Klinischer Studien”)
GLMM: Generalised linear mixed model
KC: Keratinocyte carcinoma
SCC: Squamous cell carcinoma
UVR: Ultraviolet radiation
WHO: World Health Organization
