# Supplementary material for: Increasing topical sunscreen use in German outdoor workers: results of a non-randomised controlled intervention study
Source: BMC Public Health. 2026 Sep 23;26:2656. doi: 10.1186/s12889-026-29422-6 (PMC13599276; doi:10.1186/s12889-026-29422-6)
Supplement: Supplementary file 1 [file 12889_2026_29422_MOESM1_ESM.pdf]

Sonnenschutzmittel

Allgemeine Anwendungshinweise

WANN?

- Vor der Arbeit Sonnenschutzprodukte gleichmäßig auf die **saubere und trockene Haut** auftragen.
- **Alle zwei Stunden** neu auftragen. 2h
- Das erneute Auftragen verlängert allerdings nicht die Schutzzeit, sondern stellt nur den alten, zwischenzeitlich verringerten Schutzzustand wieder her.

WIE VIEL?

- Der angegebene Schutzfaktor gilt nur, wenn **genug Sonnenschutzmittel** verwendet wird.
- Die zu verwendende Menge ist auch **abhängig von der Anwendungsform.**

WO?

- Prinzipiell gilt: jeder Teil des Körpers, der **nicht mit Kleidung bedeckt ist**, muss eingecremt werden.

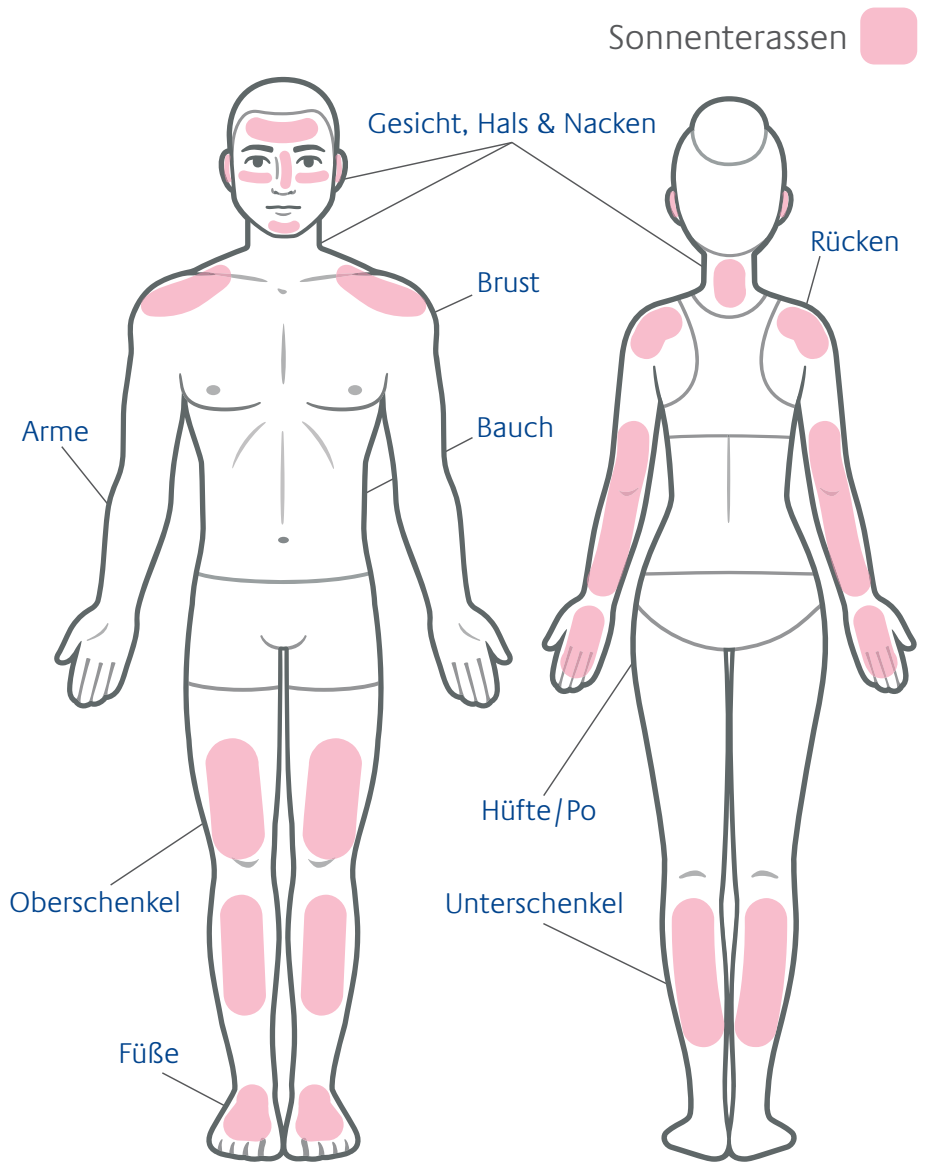

Sonnenschutzmittel

Dosiermenge pro Körperzone

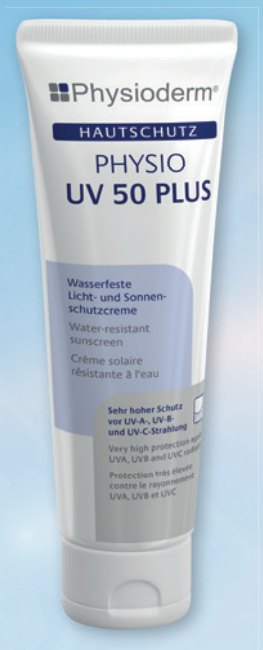

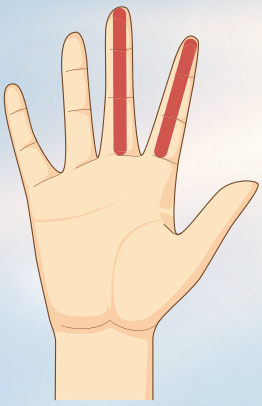

PHYSIO **UV 50 PLUS**

Gleichmäßig auf die saubere und trockene Haut auftragen. **2 Finger pro Körperzone, z. B. Gesicht, Hals und Nacken**

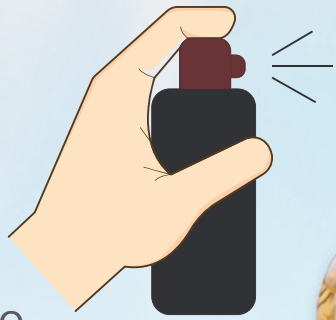

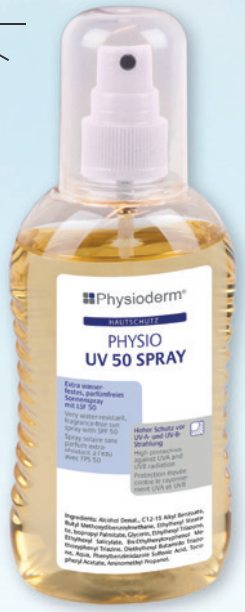

PHYSIO **UV 50 SPRAY**

Vor Gebrauch schütteln! Aus 20-30 cm Entfernung gleichmäßig und großzügig auf die Haut aufsprühen. **10 Pumpstöße pro Körperzone, z. B. Gesicht, Hals und Nacken**

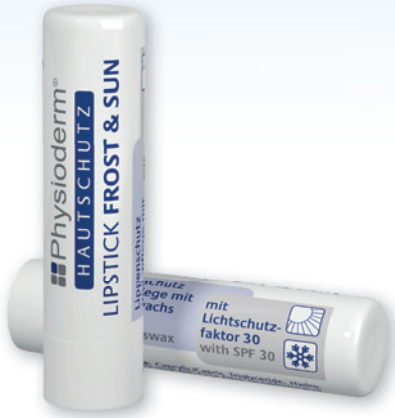

LIPSTICK **FROST & SUN**

Gleichmäßig auf den sauberen und trockenen Lippen verteilen. **Großzügig auftragen**

11-Zonen-Regel

- 2 Finger Sonnenschutzcreme bzw. 10 Pumpstöße Sonnenschutzspray pro Körperzone
- Schutz **alle 2 Stunden** neu auftragen

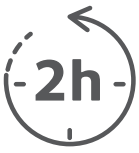

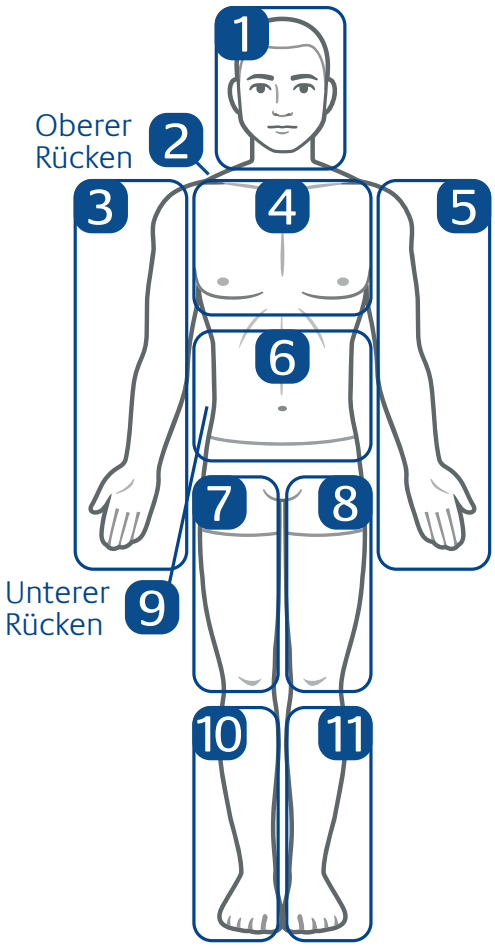

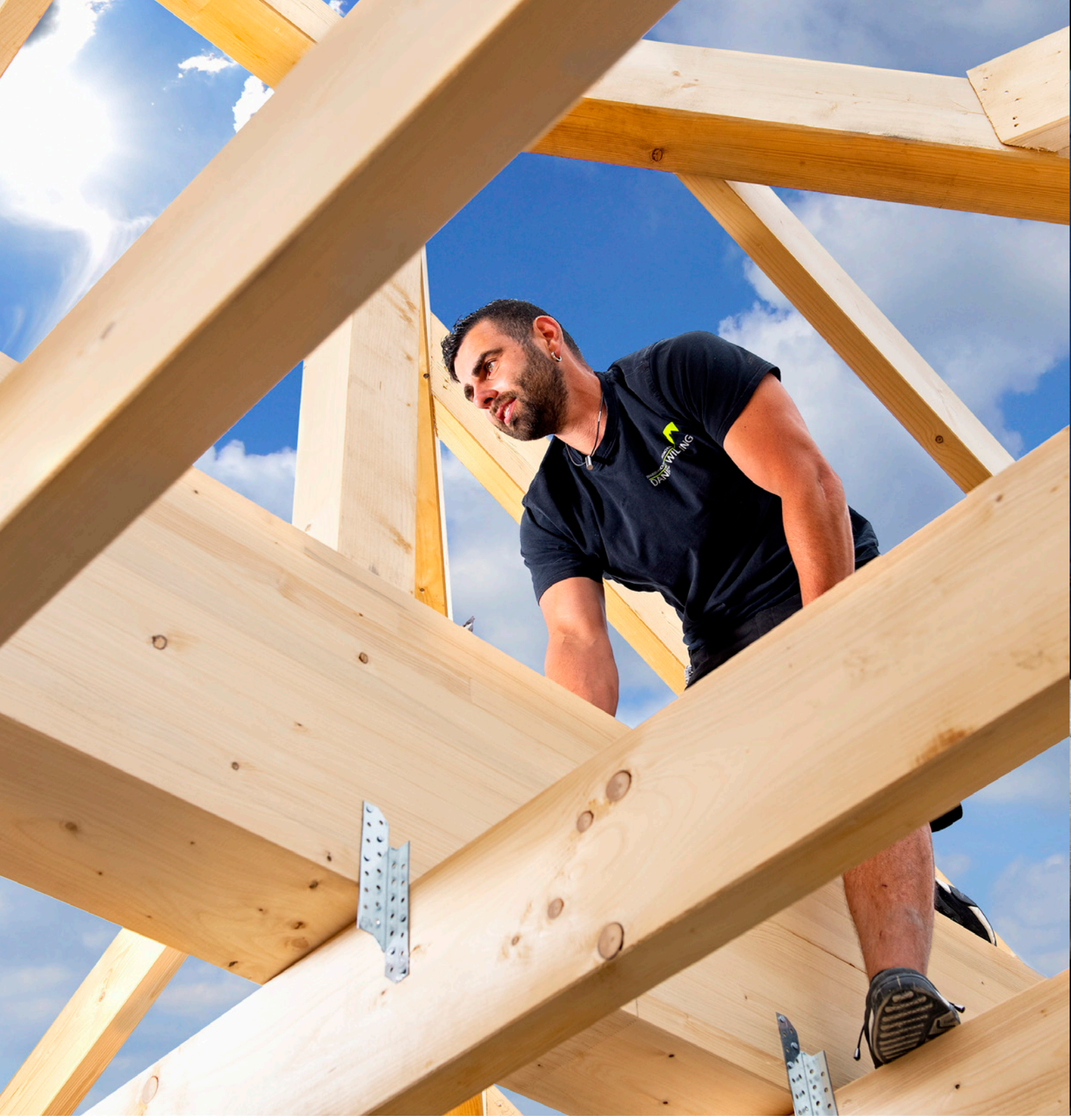

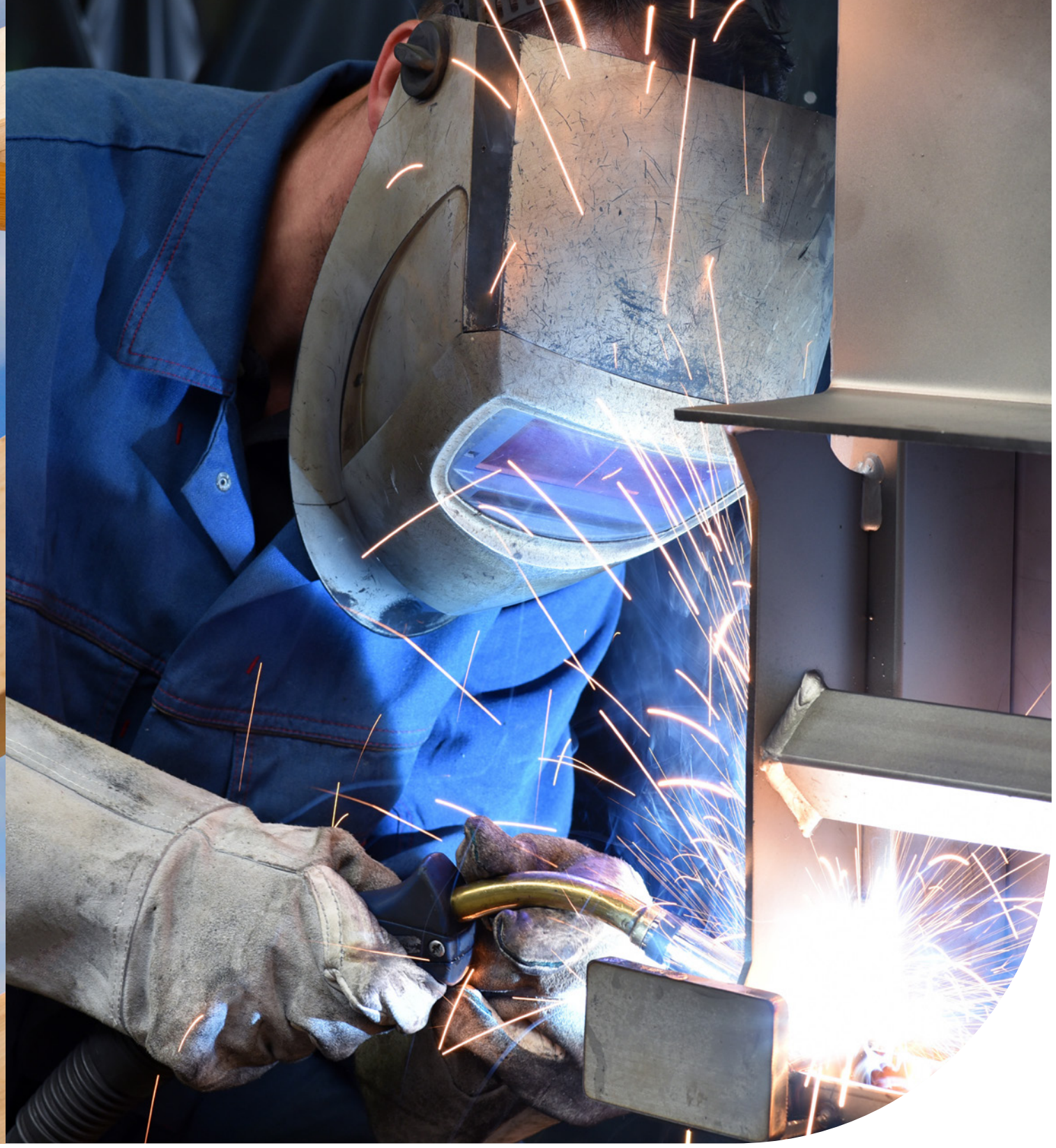

UV-Schutz am Arbeitsplatz

Hintergründe & Informationen zum professionellen Hautschutz

Your Partner for
Professional Skin Care

PGP

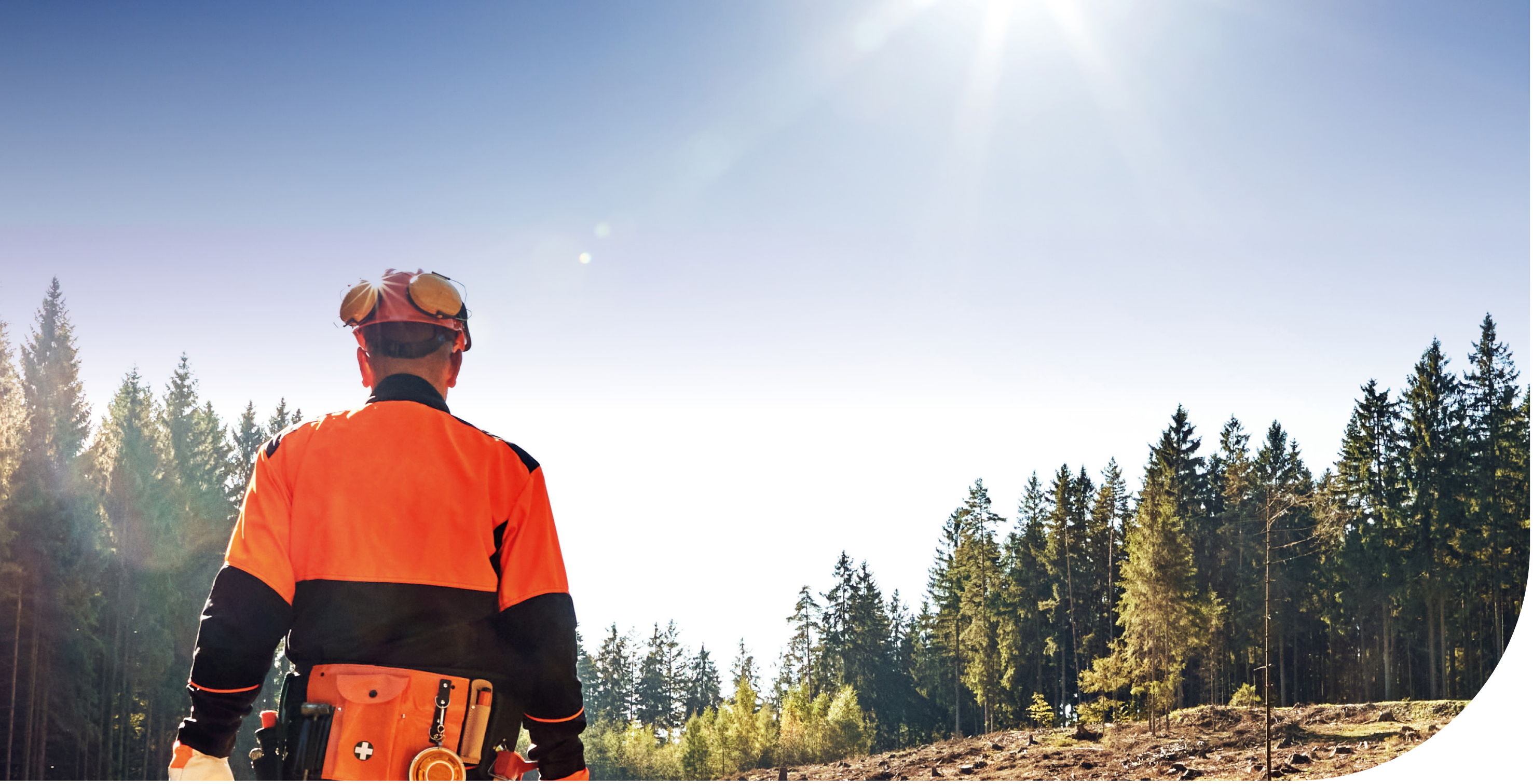

BERUFLICHER UV-SCHUTZ:
UNTER FREIEM HIMMEL UNERLÄSSLICH

Rund 7,2 Millionen Menschen arbeiten in Deutschland regelmäßig oder überwiegend im Freien und sind dadurch der deutlich erhöhten Gefahr, **an Hautkrebs zu erkranken**, ausgesetzt. Natürliches Sonnenlicht enthält UV-A- und UV-B-Strahlung, die **langfristig schwerwiegende Hautschäden** verursachen kann.

Wer unter freiem Himmel arbeitet, ist dieser Strahlung über viele Jahre hinweg regelmäßig ausgesetzt. Um dauerhafte Gesundheitsschäden zu vermeiden, ist daher ein **besonderer Schutz erforderlich**. Im schlimmsten Fall sind Dermatosen, Hautalterung und Hautkrebs die Folge.

BESONDERS GEFÄHRDETE BERUFSGRUPPEN

- Bergführer und Skilehrer
- Betonbauer
- Briefträger
- Dach- und Fassadenbauer
- Dachdecker
- Erzieher
- Garten- und Landschaftsbauer
- Kanalbauer
- Land- und Forstwirte
- Maurer
- Müllwerker
- Obst- und Gemüsegärtner
- Schweißer
- Seefahrer
- Sportlehrer
- Stahlbaumonteure
- Steinbrecher
- Straßenbauer
- Zimmerer

BERUFSKRANKHEIT WEISSER HAUTKREBS
BK 5103

Die schädlichen Auswirkungen von UV-Strahlung sind heute unumstritten. Seit 2015 ist weißer Hautkrebs als Berufskrankheit (BK-Nr. 5103) anerkannt; bereits unmittelbar nach der Anerkennung wurde er zu **einer der häufigsten gemeldeten Berufskrankheiten**. Dies hat zu einem Wandel im Umgang mit dem Thema Sonnenlicht als Risikofaktor beigetragen.

Das Bewusstsein hinsichtlich der Bedeutung von beruflichem UV-Schutz ist heute bei Arbeitgebern und Beschäftigten deutlich höher als noch vor wenigen Jahren. Arbeitgeber sind **gesetzlich verpflichtet**, alle geeigneten Maßnahmen zu ergreifen, um ihre Beschäftigten vor Berufskrankheiten zu schützen. Es gilt als erwiesen, dass bestimmte Formen des lichtbedingten weißen Hautkrebses und seine Vorstufen direkt und ausschließlich die Folge beruflicher Belastung sind.

UV-SCHUTZ IST TEIL DER PERSÖNLICHEN SCHUTZAUSRÜSTUNG (PSA)

Beruflicher Sonnenschutz ist **keine Privatsache** der Mitarbeiter. Ganz im Gegenteil: Nicht erst seit der sogenannte „weiße Hautkrebs“ eine anerkannte Berufskrankheit ist, ist ein angemessener Schutz vor UV-Strahlung für beruflich exponierte Personen Bestandteil der Persönlichen Schutzausrüstung. Arbeitgeber sind somit gesetzlich verpflichtet, jedem gefährdeten Mitarbeiter einen geeigneten Schutz zur Verfügung zu stellen.

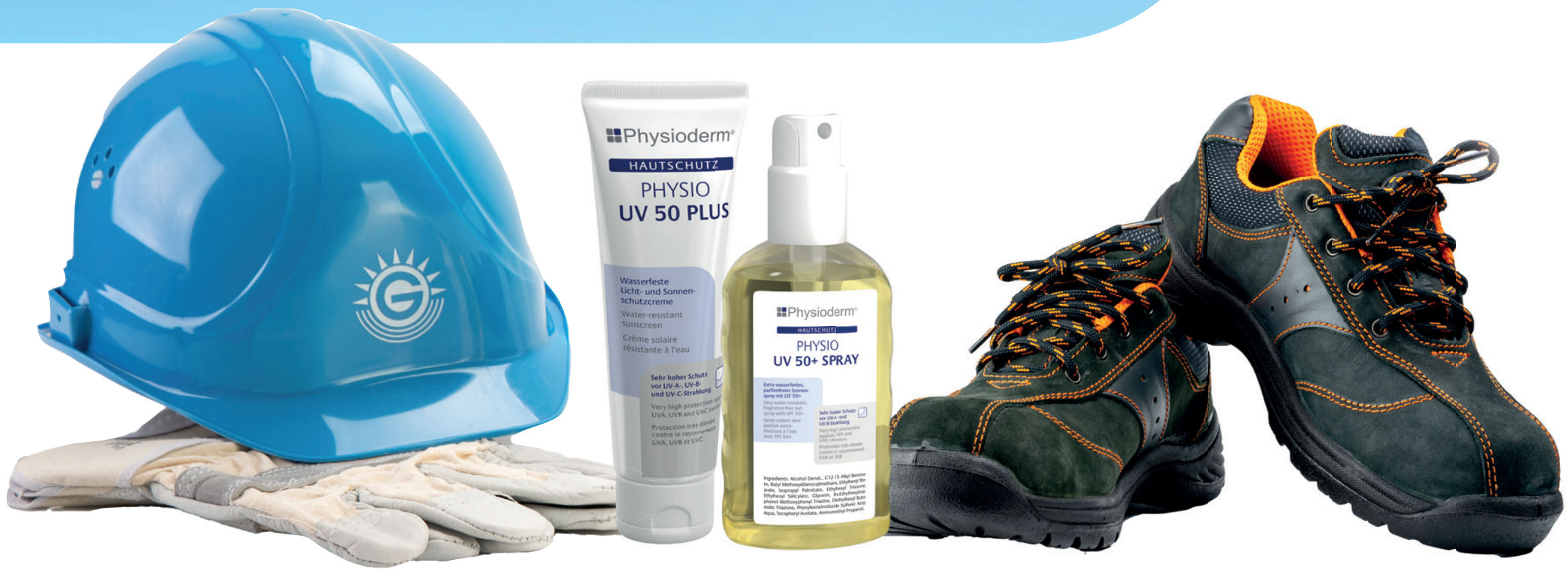

SONNENLICHT
EINE NOCH IMMER UNTERSCHÄTZTE GEFAHR

Obwohl UV-Schutz in den betroffenen Branchen und Berufen zunehmend mehr Beachtung findet, wird die Gefahr jedoch auch heute noch oftmals unterschätzt. Nicht immer sind die in Unternehmen ergriffenen Maßnahmen geeignet, Mitarbeiter effektiv zu schützen. Ebenso problematisch ist, dass Beschäftigte häufig nicht ausreichend darüber informiert sind, welche Bedeutung dem Sonnenschutz zukommt und wie sie sich wirksam vor Gesundheitsschäden durch UV-Strahlung schützen können.

UV-STRAHLEN:
AKUTE GEFÄHRDUNG AM ARBEITSPLATZ

Jeder Beschäftigte, der regelmäßig im Freien arbeitet, ist in **hohem Maße durch Sonnenlicht gefährdet**. Die unmittelbar spürbaren Gesundheitsschäden – in erster Linie Sonnenbrand und Sonnenstich – sind dabei zwar unangenehm, aber weit weniger gravierend als die drohenden **langfristigen Folgen**, wie **frühzeitige Hautalterung** und **Hautkrebs**. Auch die Augen leiden unter UV-Strahlung: Neben Horn- und Bindehautentzündungen können die erlittenen Schäden langfristig grauen Star hervorrufen.

WAS IST UV-STRAHLUNG?

Ultraviolettstrahlung – kurz UV-Strahlung – ist eine für Menschen unsichtbare elektromagnetische Strahlung, die in **natürlichem Sonnenlicht** enthalten ist. Ihre Wellenlängen von 200 bis 400 nm sind kürzer als die von sichtbarem Licht.

Die relativ langwelligen **UV-A-Strahlen** dringen tiefer in die Haut ein und sind hauptverantwortlich für eine **beschleunigte Hautalterung**. Die kurzwelligen, energiereichen **UV-B-Strahlen** sind dagegen ursächlich für das Entstehen eines Sonnenbrandes. Alle Arten der UV-Strahlung in diesem Wellenlängenbereich tragen zur **Entstehung von Hautkrebs** bei. Da die Auswirkungen der UV-A-Strahlung, im Gegensatz zum Sonnenbrand bei der UV-B-Strahlung, nicht direkt wahrgenommen werden, wird ihre Gefahr sehr häufig unterschätzt. Hinzu kommt, dass UV-A-Strahlen auch Wolken und sogar normales Fensterglas durchdringen können.

KÜNSTLICHE UV-STRAHLUNG

Die äußerst kurzwellige **UV-C-Strahlung** im Sonnenlicht wird von der Atmosphäre herausgefiltert und erreicht die Erdoberfläche nicht. Für bestimmte Berufsgruppen, wie Schweißer und Materialprüfer sind jedoch künstliche UV-C-Strahlen eine Gefahr.

Neben der direkten Strahlung kann auch die **indirekte Strahlung** (z. B. durch reflektierende oder transmittierende Oberflächen) zu einer Gefährdung führen!

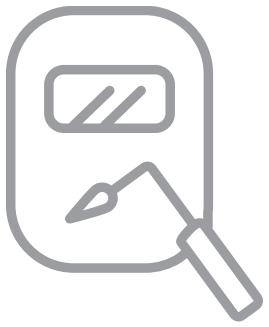

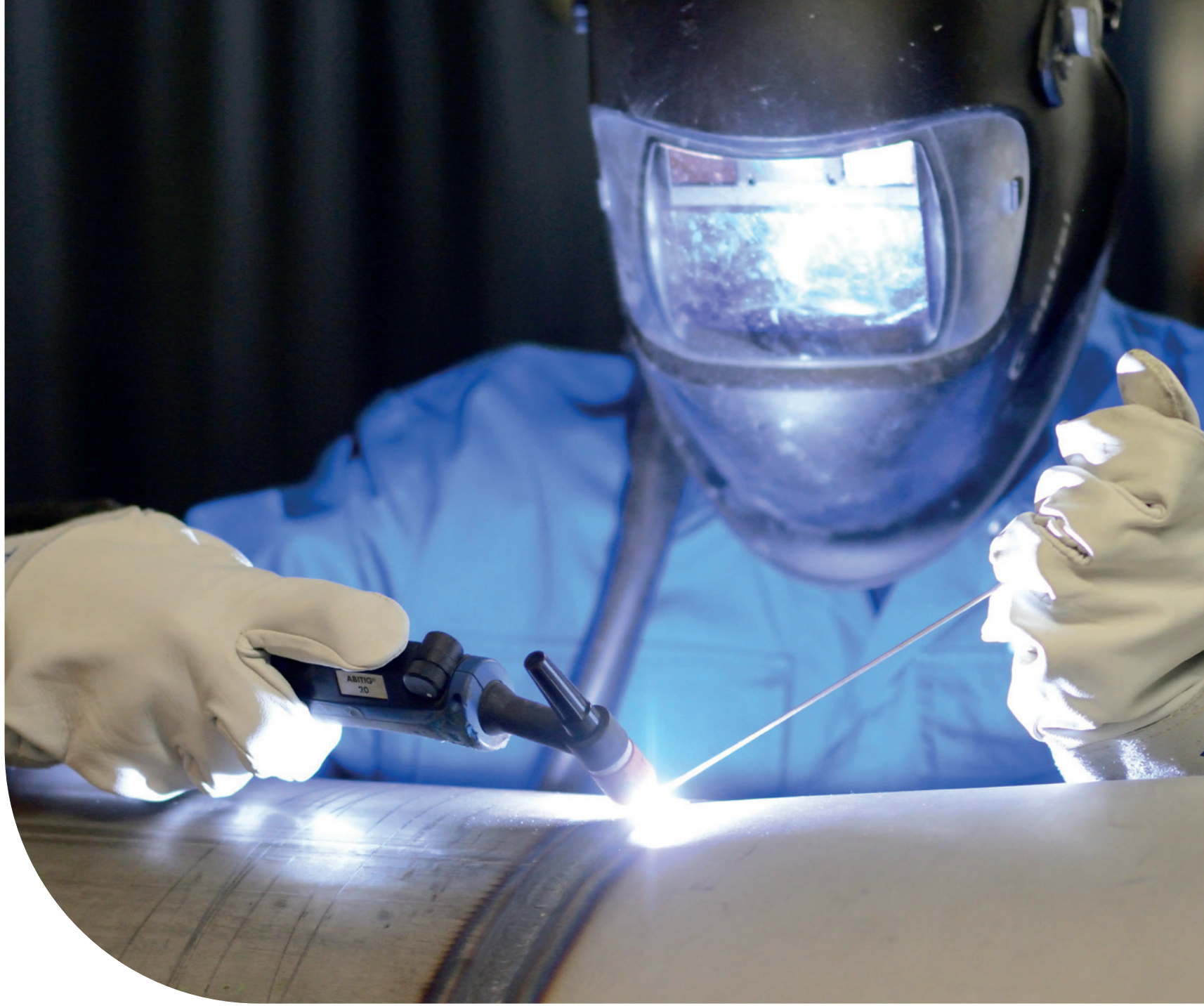

WELLENLÄNGE & AUSWIRKUNG DES SONNENLICHTS

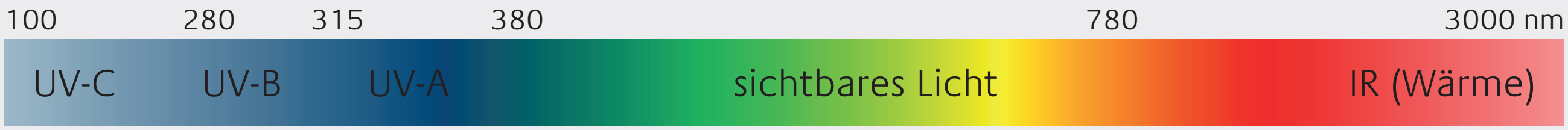

UV-C-STRAHLEN (100 – 280 NM)

- nur künstliches Vorkommen relevant
- entstehen z. B. bei Lichtschweiß-, Laser- und Elektronenstrahl-Arbeiten
- verursachen Sonnenbrand und Hautkrebs

UV-B-STRAHLEN (280 – 315 NM)

- verursachen Sonnenbrand, Hautkrebs und Horn- und Bindehautentzündung der Augen
- erforderlich für Vitamin-D-Bildung

UV-A-STRAHLEN (315 – 400 NM)

- dringen besonders tief in die Haut
- verursachen Hautkrebs, frühzeitige Hautalterung und grauen Star
- keine langfristige Bräunung, kein Eigenschutzaufbau der Haut

STARK ERHÖHTES HAUTKREBS-RISIKO BEI OUTDOOR-WORKERN

Jedes Jahr erkranken rund 200.000 Menschen in Deutschland an einer bösartigen Veränderung der Haut; weltweit sind fast 4 Millionen Menschen betroffen. Hautkrebs ist damit nicht nur die häufigste Form von Krebs, sondern eine der häufigsten Krankheiten überhaupt. UV-Strahlung gilt dabei als Hauptursache für bösartige Tumore der Haut.

KUMULATIVE SCHÄDEN DURCH LANGJÄHRIGE SONNENEINWIRKUNG

Vor allem ältere Menschen erkranken an Hautkrebs, da ihre Haut durch jahrzehntelange kumulative Schäden stark angegriffen ist. Outdoor-Worker sind in besonderem Maße gefährdet. Wer über Jahre hinweg regelmäßig unter freiem Himmel arbeitet, trägt ein doppelt so hohes Risiko an bestimmten Formen von Hautkrebs zu erkranken, wie ein Innenbeschäftigter. Eine im Vergleich um 40% höhere UV-Belastung ist dabei bereits ausreichend, um die Gefahr einer Erkrankung entsprechend zu erhöhen.

ÄUSSERST AGGRESSIV: SCHWARZER HAUTKREBS

Maligne Melanome, auch schwarzer Hautkrebs genannt, treten weit seltener auf als weißer Hautkrebs, sind jedoch wesentlich bösartiger. Auch bei dieser Hautveränderung gilt eine starke UV-Belastung als wichtigster Auslöser. Anders als bei weißem Hautkrebs, der ausschließlich auf UV-Strahlung zurückgeführt werden kann, spielt bei schwarzem Hautkrebs jedoch auch genetische Veranlagung eine Rolle. Auch wiederholte Sonnenbrände im Kindesalter sollen das Entstehen dieser Erkrankung begünstigen.

AUSSCHLIESSLICH DURCH UV-STRAHLUNG BEDINGT: WEISSER HAUTKREBS

Die häufigste Form von Hautkrebs ist der weiße oder helle Hautkrebs. Die zusammenfassende Bezeichnung umfasst sowohl Basalzellkarzinome (Basaliome) als auch die aggressiveren Plattenepithelkarzinome (Spinaliome). Beide Karzinomarten entstehen ausschließlich aufgrund der Belastung durch UV-Licht und treten meist an den so genannten Sonnenterrassen des Körpers auf, die der Strahlung in besonderem Maße ausgesetzt sind – etwa Nase, Ohren, Unterlippe, Nacken und Hände. In ihrer Frühform als aktinische Keratose zeigt sich die Erkrankung oft in Form von rauen, rötlichen Hautstellen oder einer Verdickung der Hornschicht.

WEISSER HAUTKREBS KANN FOLGE BERUFLICHER BELASTUNG SEIN

Arbeitgeber sind gesetzlich verpflichtet, alle geeigneten Maßnahmen zu ergreifen, um ihre Beschäftigten vor Berufskrankheiten zu schützen. Seit 2015 sind Plattenepithelkarzinome und multiple aktinische Keratosen der Haut als Berufskrankheit BK-Nr. 5103 anerkannt.

Es gilt als erwiesen, dass bestimmte Formen des lichtbedingten weißen Hautkrebses und seine Vorstufen direkt und ausschließlich die Folge beruflicher Belastung sind.

AKTIV GEGEN KREBS: PRÄVENTION UND FRÜHERKENNUNG

Im Kampf gegen Hautkrebs ist **Vorsorge** die beste Medizin. Neben der Prävention kommt dabei auch der rechtzeitigen Erkennung hohe Bedeutung zu, denn früh erkannter Hautkrebs ist meist gut behandelbar. Auch Menschen, die sich gut vor UV-Strahlen schützen, sollten auf auffällige Hautveränderungen achten, die ein frühes Zeichen von Hautkrebs sein können. Nicht abheilende Rötungen oder raue Stellen, Hautknötchen, unerklärliche Krusten oder Blutungen, sich verändernde Muttermale: All diese Symptome sind ein Anlass, vorsichtshalber einen Hautarzt aufzusuchen. **Regelmäßige Haut-Screenings** sind zudem generell ratsam. Ab dem 35. Lebensjahr übernehmen die gesetzlichen Krankenkassen hierfür alle zwei Jahre die Kosten.

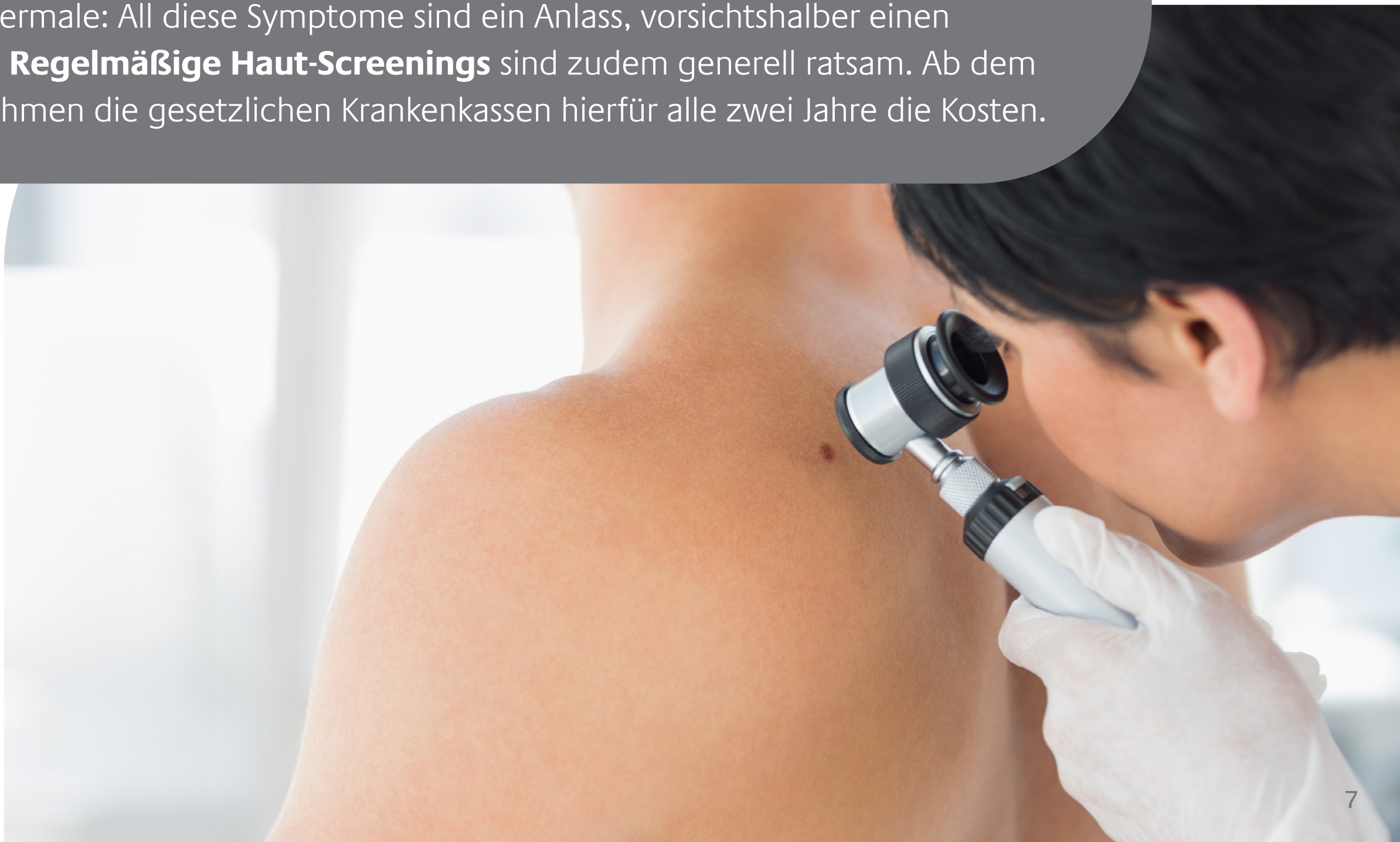

RISIKOBEWERTUNG:
UV-BELASTUNG EINSCHÄTZEN

Anders als die ebenfalls im Sonnenlicht enthaltene Infrarotstrahlung, die als Wärme gespürt wird, ist UV-Strahlung für Menschen nicht wahrnehmbar. Subjektives Empfinden ist daher beim Einschätzen des Gesundheitsrisikos durch UV-Strahlen kaum hilfreich – häufig wird die tatsächliche Gefahr unterschätzt, weil etwa der Himmel bewölkt ist oder ein kühler Wind weht. Aussagekräftige Hilfe für das Beurteilen der herrschenden UV-Belastung liefert dagegen der UV-Index, der den erwarteten Tageshöchstwert um die Mittagszeit angibt.

UV-INDEX:
PLANUNGSHILFE FÜR EFFEKTIVEN ARBEITSSCHUTZ

Der UV-Index ist eine Vorhersage der täglichen Sonnen- und UV-Belastung. Er erfasst die maximale Belastung, die an einem bestimmten Ort durch schädigende UV-Strahlen zu erwarten ist, und ist damit ein guter Anhaltspunkt für eine akkurate Risikobewertung und das entsprechende Anpassen von Arbeitsschutzmaßnahmen. Der weltweit einheitliche Index wird häufig in Wettervorhersagen angegeben und kann im Internet abgerufen werden, wo ihn unter anderem das Bundesamt für Strahlenschutz mitteilt.

www.bfs.de/DE/themen/opt/uv/uv-index/uv-index_node

UV INDEX 1, UV INDEX 2	Niedrig	Kein Schutz notwendig	Gefahrloser Aufenthalt draußen möglich.
UV INDEX 3, UV INDEX 4, UV INDEX 5	Mittel	Schutz notwendig	In der Mittagszeit Schatten suchen, T-Shirt anziehen, Sonnencreme auftragen und Kopfbedeckung tragen.
UV INDEX 6, UV INDEX 7	Hoch		
UV INDEX 8, UV INDEX 9, UV INDEX 10	Sehr hoch	Besonderer Schutz notwendig	In der Mittagszeit möglichst nicht draußen aufhalten! Unbedingt Schatten suchen! T-Shirt, Sonnenbrille, Sonnencreme und Kopfbedeckung dringend nötig.
UV INDEX 11+	Extrem		

Je höher der **UV-Index** ist, desto schneller können bei **ungeschützter Haut gesundheitliche Schäden** auftreten. In Deutschland liegt der Index üblicherweise nicht über 8. In den höheren Lagen der Alpen sind im Sommer jedoch auch Werte von 9 und 10 möglich.

ENTSCHEIDEND FÜR UV-BELASTUNG: SONNENSTAND UND HÖHE

Jahres- und Tageszeit spielen eine wesentliche Rolle für die Belastung durch UV-Strahlen. Die Intensität der Sonnenstrahlung hängt von dem Winkel ab, in dem sie auf die Erde fällt: je höher der Sonnenstand und je steiler der Einfallswinkel, desto intensiver die Strahlung. Daher ist die UV-Belastung im Sommer weit höher als im Winter und erreicht ihren Tageshöhepunkt um die Mittagszeit. Rund die Hälfte der täglichen Gesamtdosis an UV-Strahlen trifft zwischen 11 und 13 Uhr auf die Erde. Die Höhe über dem Meeresspiegel nimmt ebenfalls bedeutenden Einfluss, denn pro 1.000 Höhenmeter steigt die Strahlenintensität um etwa zehn Prozent.

RISIKOFAKTOR REFLEXION: ERHÖHUNG DER UV-BELASTUNG

Auch aus der Umgebung reflektiertes Streulicht trägt wesentlich zur Intensität der UV-Strahlung bei. Reflektierende Umgebungen wie Schnee, Wasser und heller Sand können die UV-Belastung stark erhöhen – so reflektiert etwa heller Sand bis zu 80 % der Strahlung und Schnee sogar bis zu 100 %. Selbst Wolken, die die direkte UV-Strahlung vermindern, können die tatsächliche UV-Belastung durch Reflexion kurzzeitig über das Maß hinweg erhöhen, das bei wolkenlosem Himmel herrschen würde.

WIE VIEL SONNE VERTRÄGT DIE HAUT?

	Hauttyp 1	Hauttyp 2	Hauttyp 3	Hauttyp 4
**Merkmale**	sehr helle Haut, hellblonde oder rötliche Haare	helle Haut, blonde Haare	hellbraune Haut, dunkelblonde Haare	braune Haut, schwarze Haare
**Hautreaktion auf Sonne**	immer rot, nie braun	oft rot, geringe Bräunung	mäßig braun, selten Rötungen	schnell braun, nie rot
**Eigenschutzzeit der Haut**	5 bis 10 Minuten	10 bis 20 Minuten	20 bis 30 Minuten	40 Minuten

Nicht jeder Mensch ist UV-B-Strahlen gegenüber gleichermaßen empfindlich. Je nach Hauttyp kann die Eigenschutzzeit – also die Zeitdauer, für die man die ungebräunte Haut im Laufe eines Tages maximal der Sonne aussetzen kann, ohne dass sich die Haut rötet – zwischen fünf und vierzig Minuten liegen. Outdoor-Worker, die ganz oder teilweise unter freiem Himmel arbeiten, müssen sich daher auf jeden Fall vor UV-Strahlung schützen. Die Eigenschutzzeit der Haut ist immer zu kurz, um der UV-Belastung, die durch den beruflich bedingten Aufenthalt im Freien erhöht ist, standhalten zu können. Allerdings treten unsichtbare Schäden, die zur Hautalterung und Hautkrebs führen können, schon früher auf.

PRÄVENTION DURCH GANZHEITLICHE UV-SCHUTZMASSNAHMEN

Sonnenlicht ist die häufigste krebserzeugende Einwirkung am Arbeitsplatz – für Unternehmen mit Außenbeschäftigten sollte UV-Schutz daher oberste Priorität haben. Um im Freien arbeitende Mitarbeiter vor UV-Strahlung zu schützen, ist ein ganzheitlich angelegtes Schutzkonzept erforderlich, das die jeweilige Arbeitssituation ebenso berücksichtigt wie die Gefährdungslage. Nur so kann die Gesundheit der Beschäftigten langfristig sichergestellt werden.

SUBSTITUTION ODER TECHNISCHE MASSNAHMEN

Unter Umständen kann die Gefahr durch UV-Strahlung durch ein Verlegen des Arbeitsplatzes vollständig umgangen werden. Ist eine solche Substitution nicht möglich, können eventuell technische Hilfsmittel wie Sonnenschirme und -segel, Einhausungen, Überdachungen oder geschlossene Fahrzeugkabinen die UV-Belastung reduzieren.

ORGANISATORISCHE MASSNAHMEN

Die sinnvolle Organisation von Arbeiten kann viel dazu beitragen, Beschäftigte vor UV-Strahlen zu schützen. Um die hohe UV-Belastung zwischen 11 und 13 Uhr zu umgehen, können beispielsweise Arbeits- und Pausenzeiten verschoben werden. Außenarbeiten sollten nach Möglichkeit so geplant sein, dass sie im Schatten erfolgen können – etwa immer auf der jeweils der Sonne abgewandten Gebäudeseite. Nicht zuletzt sinkt die UV-Belastung der einzelnen Beschäftigten, wenn bestimmte Arbeiten auf mehrere Mitarbeiter verteilt werden.

PERSÖNLICHE SCHUTZ-MASSNAHMEN

Kopfbedeckung mit Ohren- und Nackenschutz, UV-Schutzbrille, körperbedeckende Kleidung sowie professionelle Lichtschutzmittel zählen zu den persönlichen Schutzmaßnahmen. Als essenziell wichtige Ergänzung minimieren persönliche Maßnahmen die UV-Belastung, die nicht vollständig durch technische und organisatorische Maßnahmen umgangen werden kann. Sie sind jedoch nicht als alleiniger Schutz geeignet und können nur im Rahmen eines durchdachten Gesamtkonzepts effektiv sein.

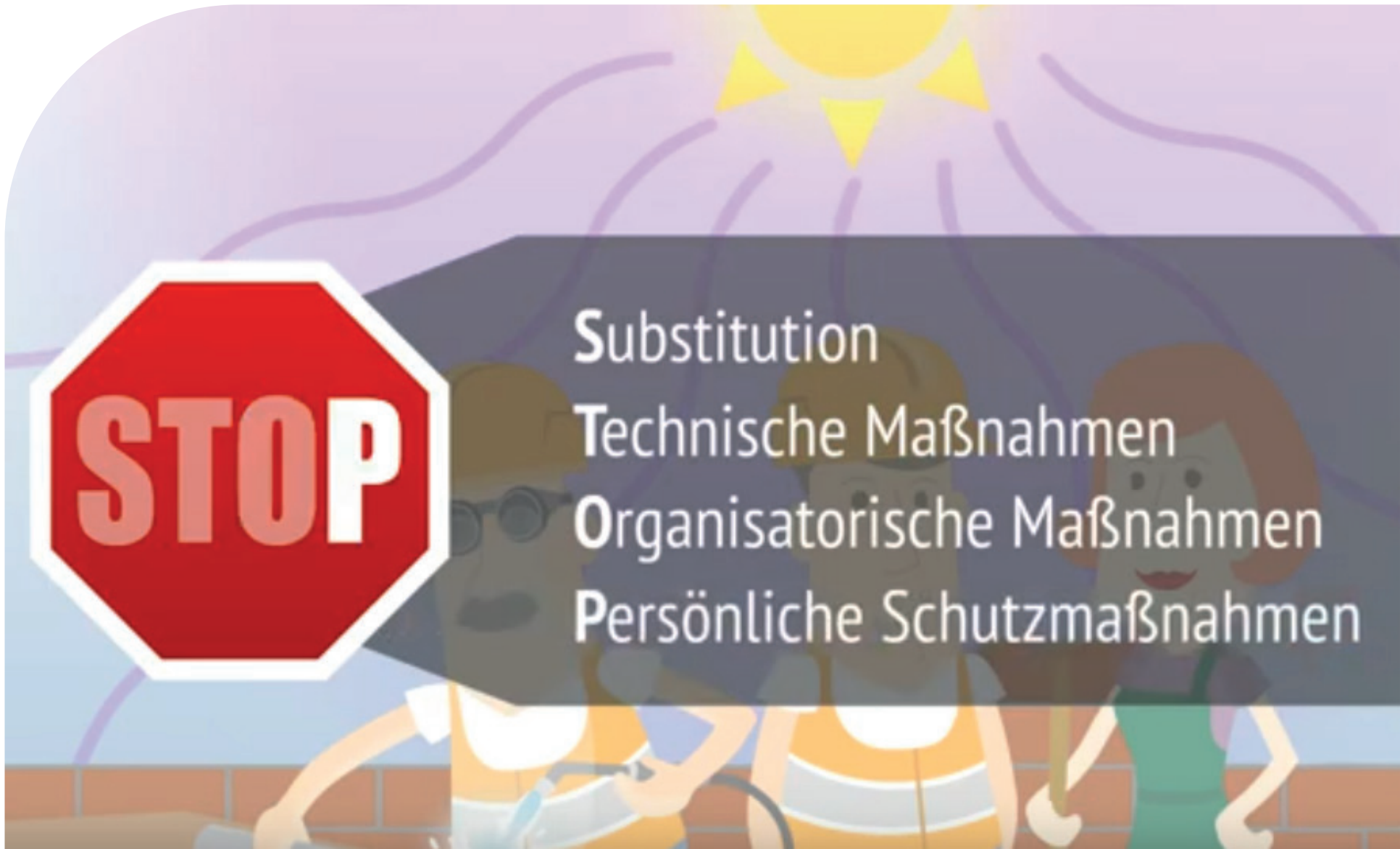

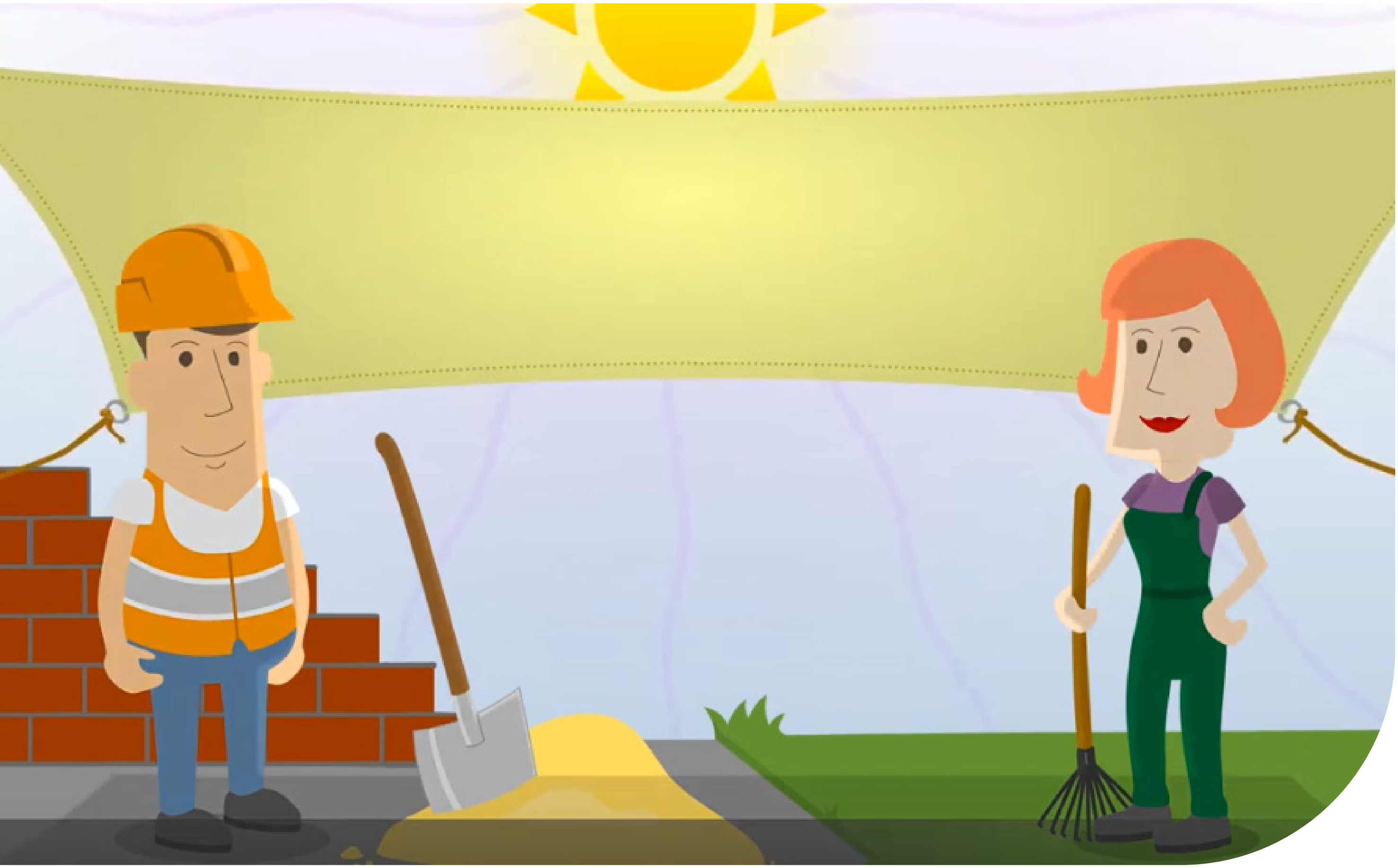

GUT INFORMIERTE MITARBEITER

Aktiv mitwirkende Beschäftigte sind eine wesentliche Voraussetzung für die Effektivität von UV-Schutzmaßnahmen. Ein wirksamer Schutz ist nur dann möglich, wenn sich jeder Mitarbeiter der Gesundheitsgefahr bewusst ist, seine persönliche UV-Belastung gezielt minimiert und die bereitgestellten Maßnahmen konsequent nutzt. In besonderem Maße gilt dies für persönliche Schutzmaßnahmen, da ihre Wirkung entscheidend von der richtigen Anwendung abhängt. Daher ist auch die Unterweisung der Mitarbeiter unerlässlich, um einen erfolgreichen UV-Schutz und das lange und gesunde Arbeitsleben der Beschäftigten sicherzustellen.

Hier Erklärvideo ansehen oder unter:
www.pgp-hautschutz.de/kompetenzcenter/videos

BERUFLICHE LICHTSCHUTZMITTEL IM FOKUS

Lichtschutzmittel sind für den wirksamen UV-Schutz am Arbeitsplatz unerlässlich. Als integraler Bestandteil eines umfassenden UV-Schutzkonzepts bieten sie Hautbereichen Schutz, die durch Kleidung nicht ausreichend geschützt werden können – meist Gesicht, Hals, Nacken und Hände. Indem sie das Eindringen von UV-Strahlung in die Haut verringern, können Lichtschutzmittel das Risiko für Hautkrebs um 40 % reduzieren. Voraussetzung für wirksamen Schutz ist, dass geeignete Produkte eingesetzt werden, die speziell für die professionelle Anwendung konzipiert sind. Ebenso wichtig: die konsequente und richtige Anwendung.

PETER GREVEN PHYSIODERM:
KOMPETENTER PARTNER FÜR PROFESSIONELLEN UV-SCHUTZ

Die richtige Auswahl von Produkten ist für den wirksamen UV-Schutz am Arbeitsplatz ebenso wichtig wie ihre konsequente und richtige Anwendung. Peter Greven Physioderm (PGP) hilft Ihnen, einen individuellen Hautschutzplan zu erstellen, der die konkreten Gegebenheiten und Schutzbedürfnisse in Ihrem Unternehmen berücksichtigt. Durch Schulungen und Beratungen vor Ort sensibilisiert PGP Ihre Mitarbeiter für das Thema und vermittelt die Anwendung und Wirkung von UV-Schutzprodukten bei Außenarbeiten.

ANWENDUNG ALS ENTSCHEIDENDER SCHUTZFAKTOR

Ein kritischer Faktor für die Wirksamkeit von Lichtschutzmitteln ist ihre Anwendung. Sie müssen großzügig aufgetragen und regelmäßig erneuert werden – nur dann können sie ihre Schutzwirkung entfalten. Der Lichtschutzfaktor gibt an, um welchen Wert das Produkt die Eigenschutzzeit der Haut verlängert: So erlaubt ein Produkt mit LSF 30 Beschäftigten, sich dreißigmal länger der Sonne auszusetzen, ohne Schaden durch UVB-Strahlen zu erleiden. Der Schutzfaktor gilt jedoch nur, wenn pro Quadratzentimeter Haut mindestens 2 Milligramm Schutzmittel verwendet werden. In der Praxis tragen Beschäftigte oft deutlich weniger auf, was die Wirkung reduzieren oder ganz verhindern kann. Darüber hinaus verlieren Sonnenschutzmittel mit der Zeit durch Reibung, Schweiß und andere Einflüsse an Wirkung und müssen rund alle zwei Stunden neu aufgetragen werden. Das erneute Auftragen verlängert allerdings nicht die Schutzzeit, sondern stellt nur den alten, zwischenzeitlich verringerten Schutzzustand wieder her.

UV-SCHUTZ
EMPFOHLENE DOSIERMENGE PRO KÖRPERZONE

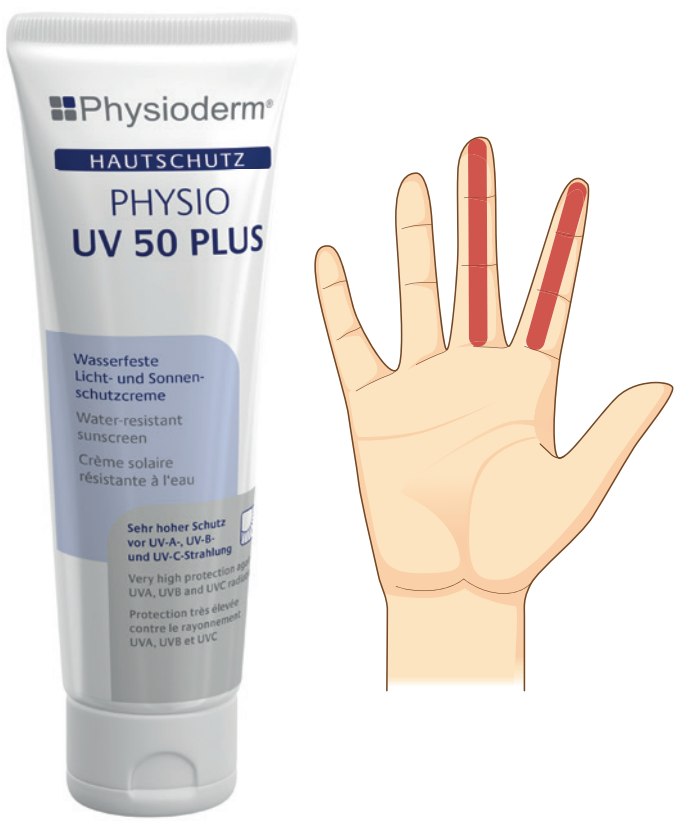

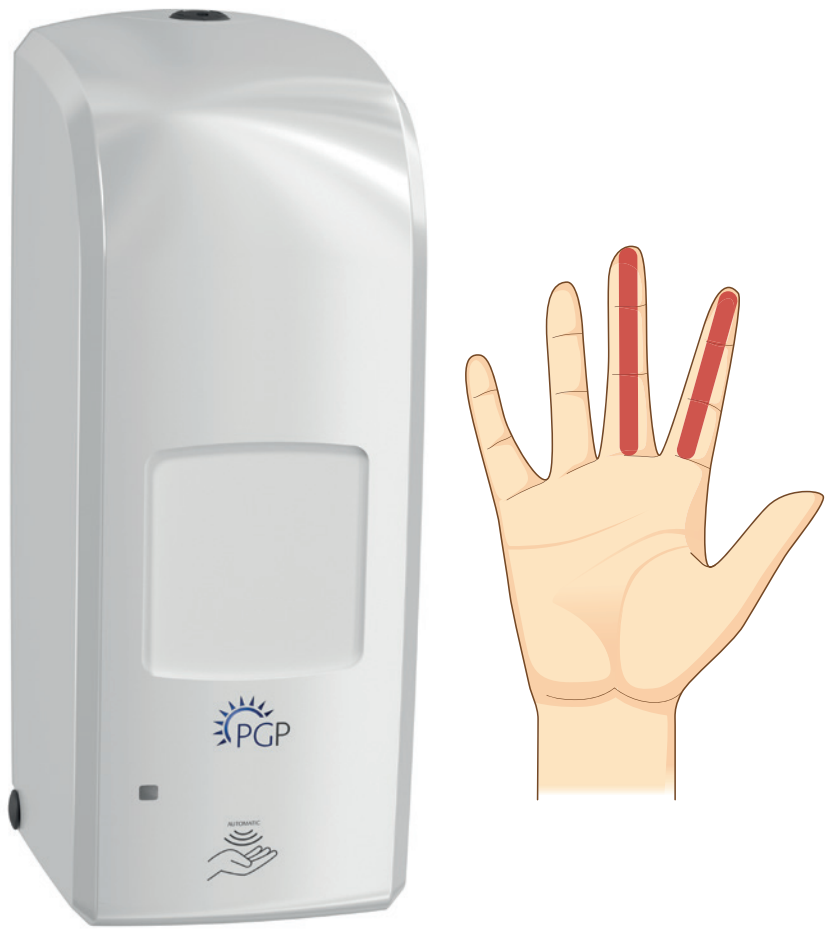

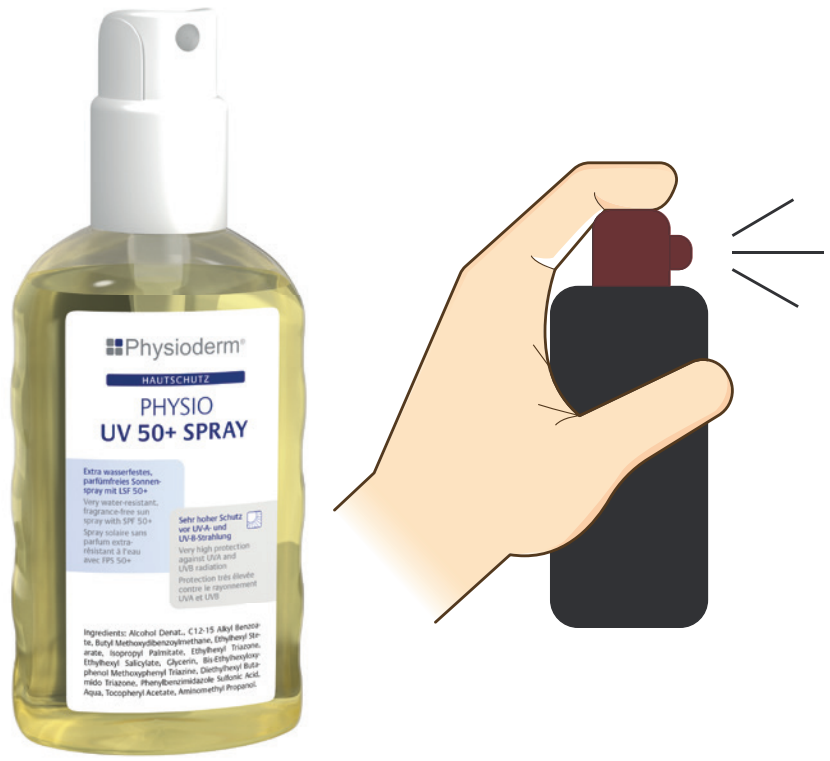

TUBE

Benötigte Produktmenge pro Körperzone: Länge des Zeige- und Mittelfingers.

SPENDERSYSTEM

Benötigte Produktmenge pro Körperzone: Spender 3 mal betätigen.

SPRAY

Benötigte Produktmenge pro Körperzone: Sprühkopf 10 mal drücken.

Körperzonen

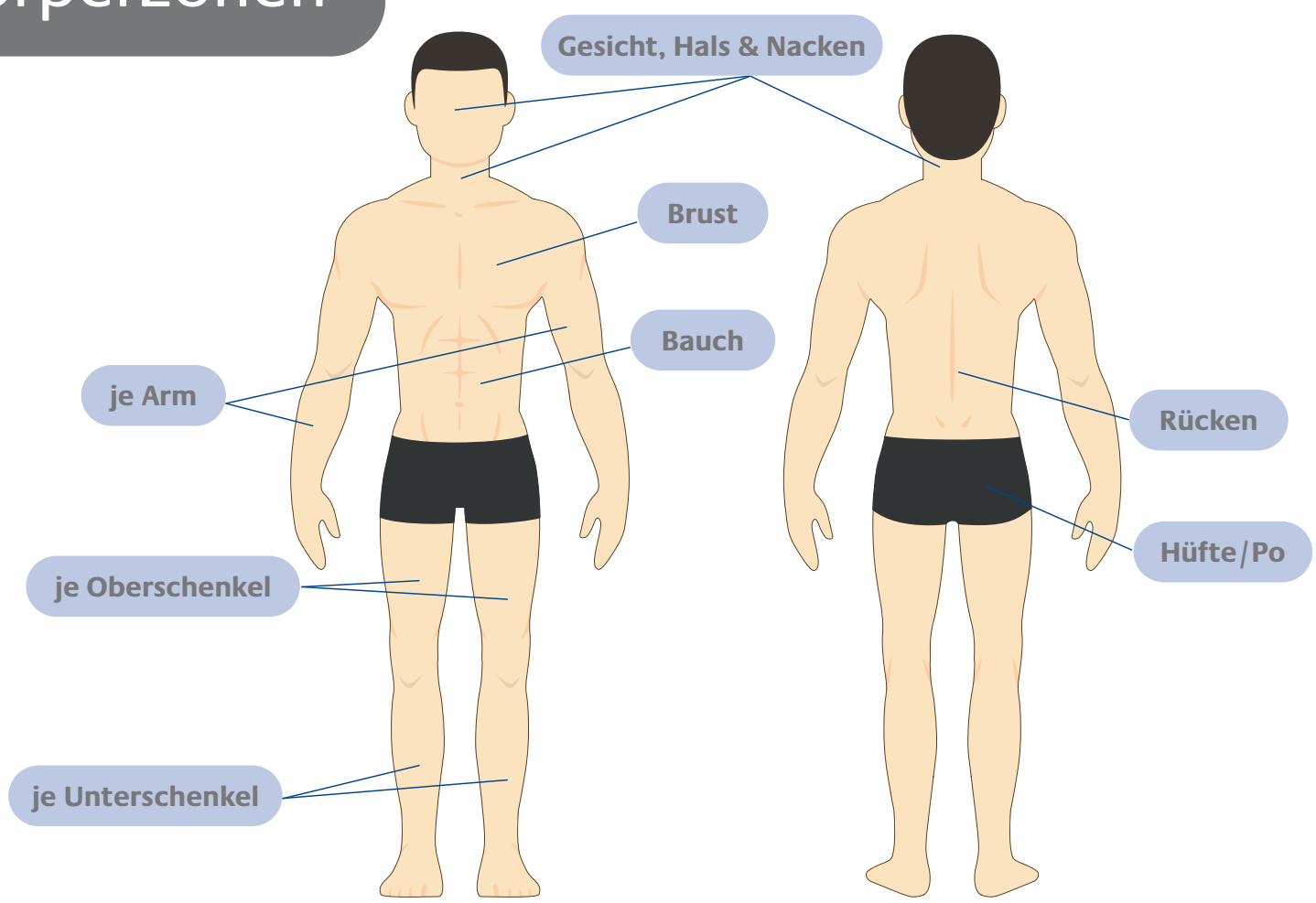

SONNENSCHUTZMITTEL RICHTIG ANWENDEN

- Großzügig auftragen: 2 mg/cm^2 (ca. einen Teelöffel für Gesicht, Hals und Ohren)
- Faustregel: pro Körperzone eine Menge verwenden, die zwei Finger großzügig bedeckt
- Regelmäßig erneuern: mindestens alle zwei Stunden oder nach Bedarf

PROFESSIONELLER UV-SCHUTZ MIT PGP

Hautschutzprofi Peter Greven Physioderm (PGP) hält alles bereit, was Arbeitgeber für den wirksamen UV-Schutz am Arbeitsplatz benötigen. Durch umfassende Dienstleistungen und professionelle Produkte unterstützt der Experte Unternehmen dabei, jedem gefährdeten Beschäftigten optimalen Schutz zu bieten. Gefährdungsbeurteilungen und passgenaue Schutzkonzepte zählen dabei ebenso zu PGPs Leistungsangebot wie Informationsmaterial und Schulungen, die Mitarbeitern vor Ort die richtige Anwendung von UV-Schutzmitteln vermitteln. Nicht zuletzt bietet PGP **hochwertige Lichtschutzmittel**, die genau auf die Anforderungen im professionellen Bereich abgestimmt sind.

EIGENSCHAFTEN EINES GUTEN BERUFLICHEN LICHTSCHUTZMITTELS AUF EINEN BLICK

- Zuverlässiger Schutz vor UV-A- und UV-B-Strahlen. Zusätzlich vor UV-C-Strahlen beim Umgang mit künstlichen UV-Quellen
- Hoher LSF: mindestens 30, im optimalen Fall 50+
- Möglichst ausgeglichener UV-A- und UV-B-Schutz
- Hohe Wasserfestigkeit mit gutem Einzugsverhalten
- Verzicht auf möglicherweise allergene Inhaltsstoffe, wie z. B. Parfüm
- Keine negative Beeinträchtigung von Werkstoffen und Arbeitsprozessen

PROFESSIONELLE PRODUKTE FÜR DEN BERUFLICHEN SCHUTZ

UV-Schutzmittel für die Verwendung am Arbeitsplatz müssen besonders hohe Anforderungen erfüllen. Hochwertige professionelle Produkte verhindern nicht nur einen Sonnenbrand, sondern bieten auch zuverlässigen Schutz gleichermaßen vor UV-B- und UV-A-Strahlung[1)] und bei Bedarf sogar vor schädlichen UV-C-Strahlen. Colipa empfiehlt ein Verhältnis von 1:3 für UV-A- zu UV-B-Schutz[2)]. In Anbetracht der Länge, die ein Outdoor-Worker der Strahlung ausgesetzt ist, und der zu geringen Auftragsmenge sollte jedoch ein höherer UV-A-Schutz bevorzugt werden. Ein ausgeglichener, sogenannter homöostatischer Schutz würde 5 Sternen im britischen Boots Star Rating entsprechen[3)]. Professionelle Produkte sind außerdem frei von Duftstoffen und idealerweise extra wasserfest. So widerstehen sie selbst starkem Schwitzen und verringern das Risiko allergischer Reaktionen auch bei langfristiger Anwendung. Außerdem dürfen Produkte für den professionellen Bereich nachweislich keine Arbeitsprozesse negativ beeinträchtigen.

Quellen: 1) Uli Osterwalder, Prävention Aktuell 03/2019 2) https://www.bvl.bund.de/DE/Arbeitsbereiche/03_Verbraucherprodukte/02_Verbraucher/03_Kosmetik/06_Sonnenschutzmittel/bgs_kosmetik_sonnenschutzmittel_node.html; STANDARDISATION MANDATE M/389 der Europäischen Kommission und Colipa 3) https://www.boots.com/sun-and-holiday-inspiration/suncare-advice/spf-factors-uva-and-uvb

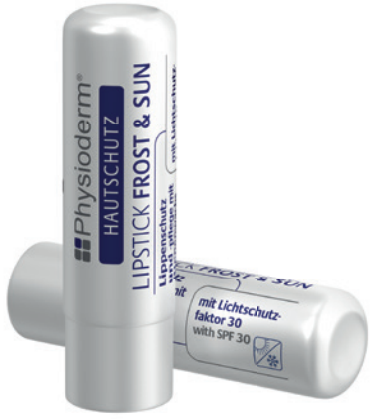

Physioderm®
LIPSTICK FROST & SUN

Lippenschutz und -pflege bei Kälte, Wind und trockener Luft sowie zum Schutz vor natürlicher UV-Strahlung.

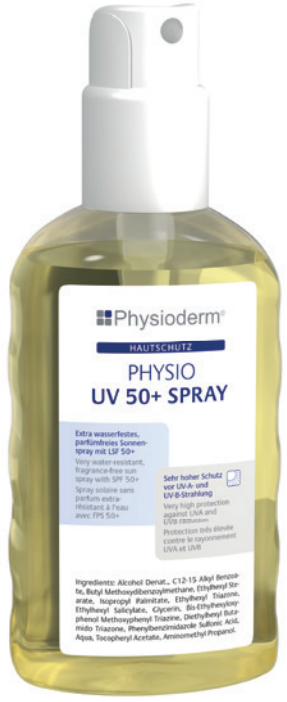

Physioderm®
PHYSIO UV 50+ SPRAY

Parfümfreies Sonnenspray zum sofortigen Schutz vor hoher natürlicher UV-Strahlung bei Arbeiten im Freien.

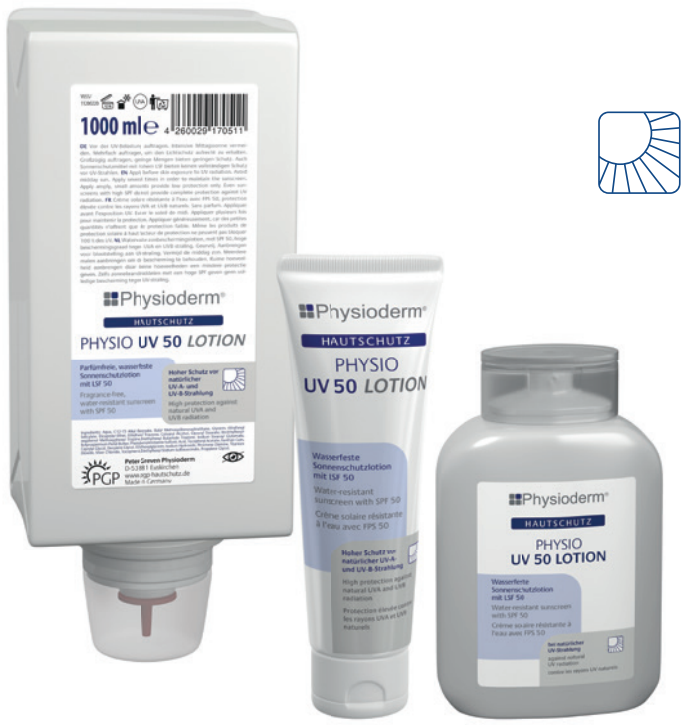

Physioderm®
PHYSIO UV 50 LOTION

Sonnenschutzlotion mit LSF 50. Hoher Schutz vor natürlicher UV-A-und UV-B-Strahlung.

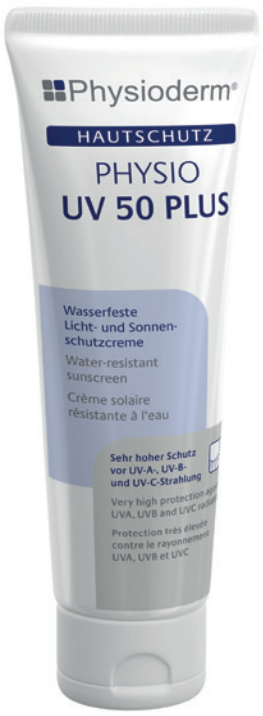

Physioderm®
PHYSIO UV 50 PLUS

Licht- und Sonnenschutzcreme bei Arbeiten im Freien unter extremen Bedingungen, bei Schweißarbeiten und beim Umgang mit Photosensibilisatoren.

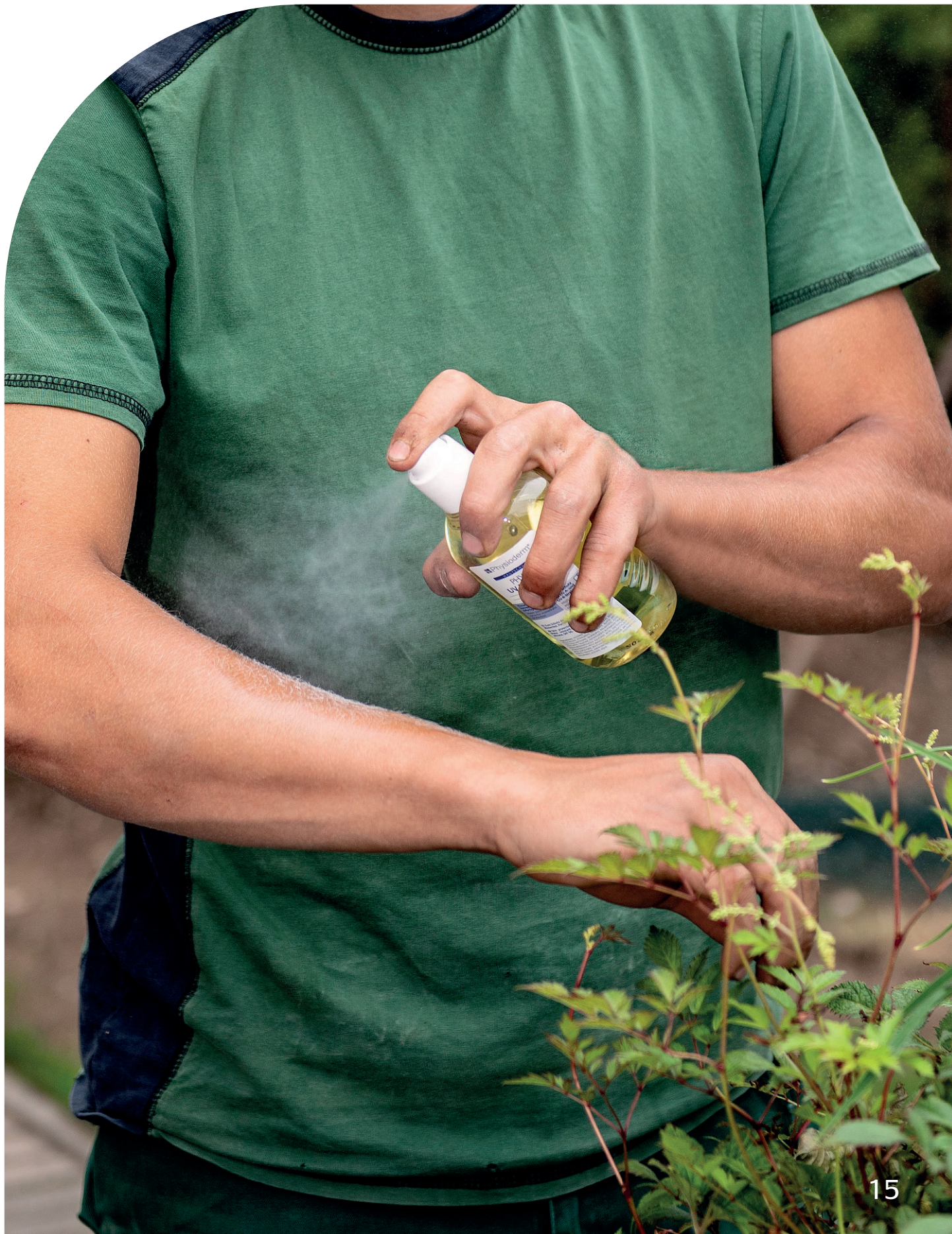

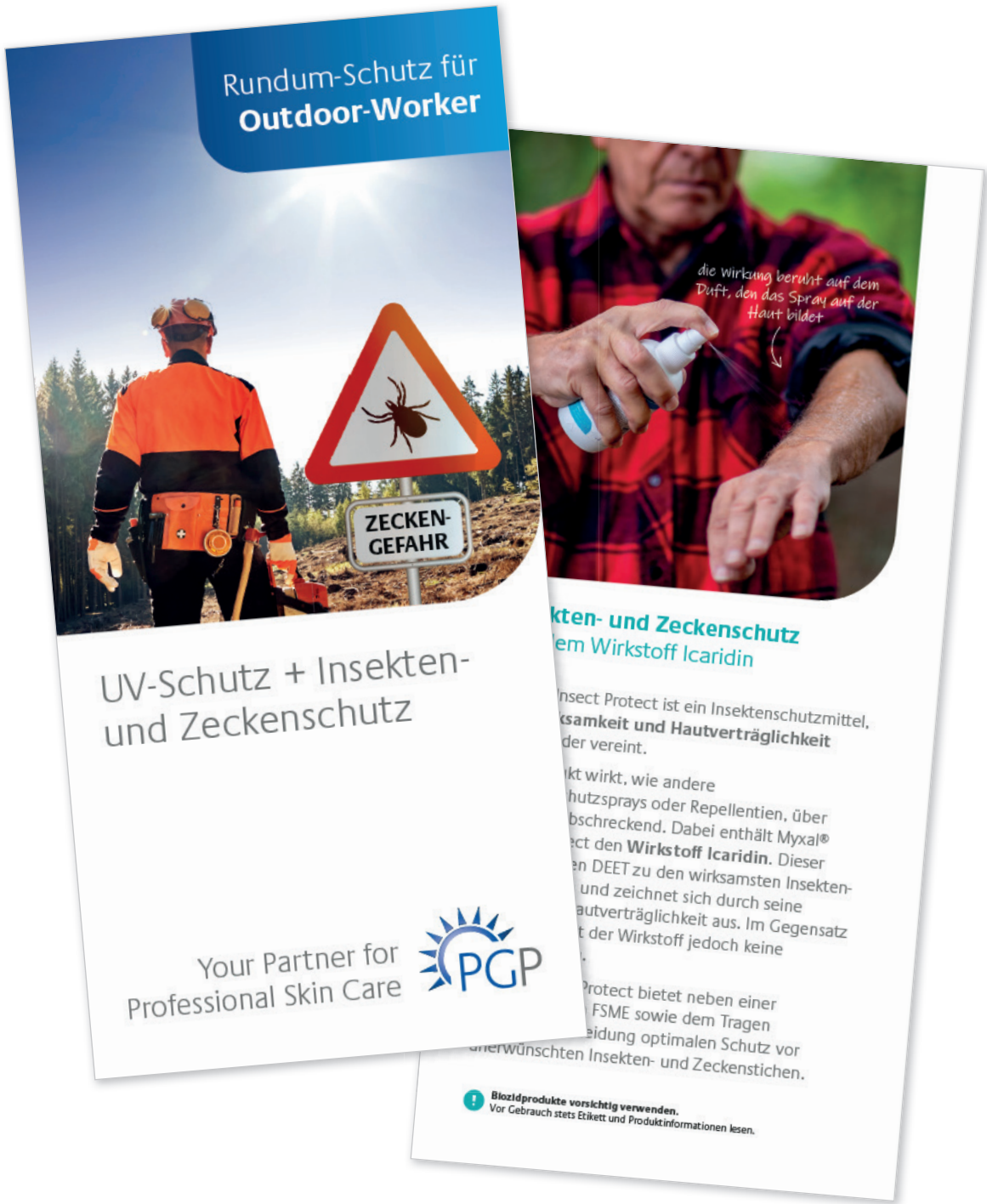

Rundum-Schutz für **Outdoor-Worker**

Neben unseren UV-Schutz-Produkten bieten wir auch ein Insekten- und Zeckenschutzmittel an.

Weitere Informationen erhalten Sie im Flyer auf unserer Webseite:

www.pgp-hautschutz.de/broschueren/flyer

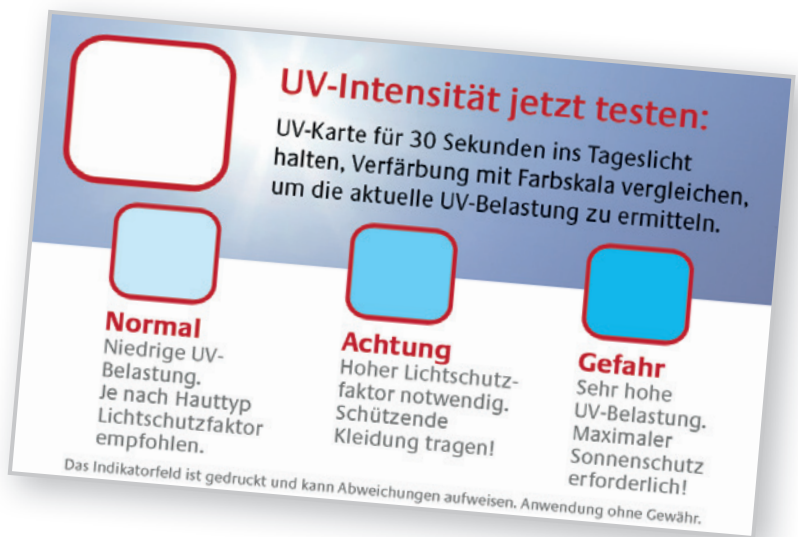

UV-Schutzkärtchen zeigen die aktuelle Intensität der lokalen UV-Belastung an. Wir stellen Unternehmen dieses praktische Hilfsmittel gerne zur Verfügung.

05/2026_11111009

Peter Greven Physioderm GmbH
Procter-&-Gamble-Straße 26
D-53881 Euskirchen

Telefon: +49 (0)2251 77617-0
info@pgp-hautschutz.de
www.pgp-hautschutz.de
