# Supplementary material for: Increasing topical sunscreen use in German outdoor workers: results of a non-randomised controlled intervention study
Source: BMC Public Health. 2026 Sep 23;26:2656. doi: 10.1186/s12889-026-29422-6 (PMC13599276; doi:10.1186/s12889-026-29422-6)
Supplement: Supplementary file 2 [file 12889_2026_29422_MOESM2_ESM.pdf]

Sonnenschutz-Fragebogen

Geburtsdatum: ____________

Geschlecht (m/w/d): ____

Bitte beantworten Sie folgende Fragen hinsichtlich Ihres Sonnenschutzverhaltens bei der Arbeit.

1. Welche berufliche Tätigkeit üben Sie aus?

2. Wie lange üben Sie bereits diese Tätigkeit aus?				
< 1 Jahr	1 – 5 Jahre	6 – 10 Jahre	11 – 20 Jahre	> 20 Jahre
☐	☐	☐	☐	☐

3. Wie viele Stunden pro Woche üben Sie diese Tätigkeit aus (*im Durchschnitt*)?	
	Stunden pro Woche

4. Wie viele Stunden pro Tag verbringen Sie während einem gewöhnlichen Arbeitstag im Freien (*zusammengerechnet über den Tag*)?			
< 2 Stunden	*2 – 4 Stunden*	*5 – 6 Stunden*	*> 6 Stunden*
☐	☐	☐	☐

5. Wie häufig verwenden Sie **allgemein** topische Sonnenschutzmittel (*Sonnencreme etc.*)?				
nie	*selten*	*manchmal*	*überwiegend*	*immer*
☐	☐	☐	☐	☐

6. Wie häufig verwenden Sie **<u>bei der Arbeit</u>** topische Sonnenschutzmittel (*Sonnencreme etc.*)?				
nie	*selten*	*manchmal*	*überwiegend*	*immer*
☐	☐	☐	☐	☐

7. Wie häufig verwenden Sie **<u>privat</u>** topische Sonnenschutzmittel (*Sonnencreme etc.*)?				
nie	*selten*	*manchmal*	*überwiegend*	*immer*
☐	☐	☐	☐	☐

8. Wie oft verwenden Sie an einem **sonnigen** Arbeitstag topische Sonnenschutzprodukte (*z. B. Sonnencreme*)?			
gar nicht	morgens einmal	morgens + nach der Mittagspause einmal	alle zwei Stunden und bei Bedarf
☐	☐	☐	☐

9. Wie oft verwenden Sie an einem **bewölkten** Arbeitstag topische Sonnenschutzprodukte (*z. B. Sonnencreme*)?			
gar nicht	morgens einmal	morgens + nach der Mittagspause einmal	alle zwei Stunden und bei Bedarf
☐	☐	☐	☐

10. Welche Sonnenschutzprodukte verwenden Sie und welchen Lichtschutzfaktor (LSF) haben diese?	
Produktname inkl. LSF *(Arbeit)*	
Produktname inkl. LSF *(privat)*	
☐	*Ich verwende keine Sonnenschutzprodukte.*

11. Wie oft hatten Sie im vergangenen Zeitraum von April bis September einen Sonnenbrand?	
	Mal

Vielen Dank für Ihre Unterstützung!
